# Drug Discovery in the Field of β-Lactams: An Academic Perspective

**DOI:** 10.3390/antibiotics13010059

**Published:** 2024-01-08

**Authors:** Lian M. C. Jacobs, Patrick Consol, Yu Chen

**Affiliations:** Department of Molecular Medicine, Morsani College of Medicine, University of South Florida, Tampa, FL 33612, USA; lianj@usf.edu (L.M.C.J.); patrickconsol@usf.edu (P.C.)

**Keywords:** β-lactamase, β-lactam, β-lactamase inhibitor, resistance mechanisms, peptidoglycan, cell wall transpeptidase

## Abstract

β-Lactams are the most widely prescribed class of antibiotics that inhibit penicillin-binding proteins (PBPs), particularly transpeptidases that function in peptidoglycan synthesis. A major mechanism of antibiotic resistance is the production of β-lactamase enzymes, which are capable of hydrolyzing β-lactam antibiotics. There have been many efforts to counter increasing bacterial resistance against β-lactams. These studies have mainly focused on three areas: discovering novel inhibitors against β-lactamases, developing new β-lactams less susceptible to existing resistance mechanisms, and identifying non-β-lactam inhibitors against cell wall transpeptidases. Drug discovery in the β-lactam field has afforded a range of research opportunities for academia. In this review, we summarize the recent new findings on both β-lactamases and cell wall transpeptidases because these two groups of enzymes are evolutionarily and functionally connected. Many efforts to develop new β-lactams have aimed to inhibit both transpeptidases and β-lactamases, while several promising novel β-lactamase inhibitors have shown the potential to be further developed into transpeptidase inhibitors. In addition, the drug discovery progress against each group of enzymes is presented in three aspects: understanding the targets, screening methodology, and new inhibitor chemotypes. This is to offer insights into not only the advancement in this field but also the challenges, opportunities, and resources for future research. In particular, cyclic boronate compounds are now capable of inhibiting all classes of β-lactamases, while the diazabicyclooctane (DBO) series of small molecules has led to not only new β-lactamase inhibitors but potentially a new class of antibiotics by directly targeting PBPs. With the cautiously optimistic successes of a number of new β-lactamase inhibitor chemotypes and many questions remaining to be answered about the structure and function of cell wall transpeptidases, non-β-lactam transpeptidase inhibitors may usher in the next exciting phase of drug discovery in this field.

## 1. Introduction

**Figure 1:**
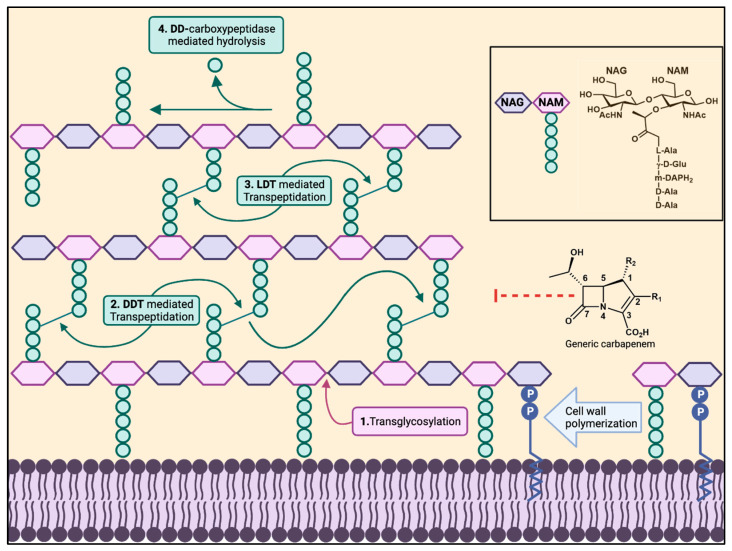
Bacterial cell wall biosynthesis. The polymerization of a bacterial cell wall peptidoglycan layer consists of transglycosylation (1), transpeptidation (2,3). _D,D_-Carboxypeptidases cleave between the last two D-alanines of the pentapeptide (4), shortening it to a tetrapeptide. _D,D_-Transpeptidases and _L,D_-transpeptidases create the 4→3 and 3→3 transpeptide linkages, respectively, cleaving the terminal D-alanine in the pentapeptide or tetrapeptide. The box on the right shows the peptide composition of NAM in Gram-negative bacteria and Gram-positive bacilli.

β-Lactam antibiotics contain a four-membered azetidinone ring and are divided into four classes: penicillins, cephalosporins, carbapenems/penems, and monobactams [1]. These antibiotics target bacterial cell wall transpeptidases, which are enzymes that crosslink cell wall pentapeptides during peptidoglycan synthesis. The β-lactam ring covalently inhibits the _D,D_-transpeptidase activity of penicillin-binding proteins (PBPs) through acylation of the enzyme’s catalytic serine, resulting in a stable acyl-enzyme complex that prevents the formation of the 4→3 transpeptide cross links (Figure 1) [2,3]. β-Lactams, specifically carbapenems, can also target _L, D_-transpeptidases (Ldts), structurally distinct enzymes responsible for creating 3→3 transpeptide cross-links via a catalytic cysteine [4,5]. 

**Figure 2:**
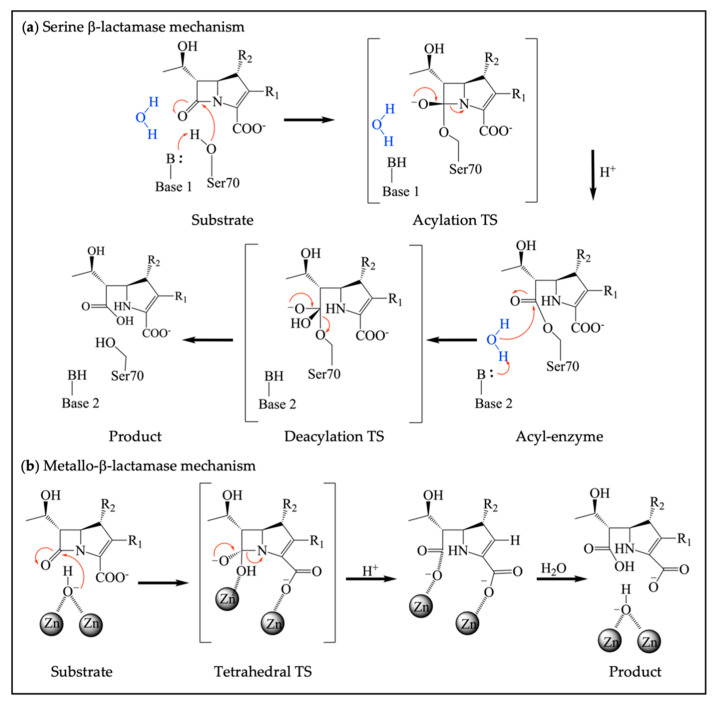
Different mechanisms of serine β-lactamases and metallo-β-lactamases. (**a**) Catalytic mechanism of SBLs, where the catalytic serine forms an acyl-enzyme intermediate with H^+^ provided by a general acid, followed by a deacylation step to release a hydrolyzed β-lactam; (**b**) Catalytic mechanism of MBLs, which rely on coordinated zinc ions for the activation of a nucleophilic water to hydrolyze substrate. H^+^ is from the solution.

The use of β-lactams to treat infection has faced multiple mechanisms of resistance by bacteria, including decreasing influx by downregulating porins, activating efflux pumps, modifying the target, and inactivating the antibiotic by enzymatic degradation [6]. For Gram-positive bacteria, β-lactam-insensitive PBPs and Ldts, encoded by mobile genetic elements or chromosomes, enable the bacteria to circumvent inhibition by these antibiotics [7]. The most prevalent means of resistance, especially for Gram-negative bacteria, occurs via enzymatic degradation involving β-lactamase enzymes [8]. β-Lactamases hydrolyze the amide bond of the azetidinone ring, preventing the β-lactam from targeting PBPs. β-Lactamases are categorized into four molecular classes: classes A, C, and D are serine β-lactamases (SBL), while class B enzymes are metallo-β-lactamases (MBL), which are further divided into three subclasses. The three classes of serine β-lactamases differ based on their overall sequence and the residues that act as the general base in catalysis, yet all contain a nucleophilic serine that attacks the carbonyl carbon of the β-lactam amide bond, creating an acyl-enzyme intermediate (Figure 2a). The deacylation step involves the nucleophilic attack from a water molecule that hydrolyzes the acyl-enzyme, releasing the degraded β-lactam product. Both the acylation and deacylation steps proceed through a tetrahedral high-energy state. In comparison, MBLs use at least one coordinated zinc ion within their active site that can activate a water nucleophile for hydrolysis (Figure 2b) [9,10,11]. The reaction also involves a tetrahedral transition state (TS) without the formation of any covalent intermediates.

Throughout the years, cell wall transpeptidases and β-lactamases have provided valuable model systems for academic researchers to study enzyme structure, function, and inhibition. In the field of drug discovery, significant strides have been made in understanding these important antibiotic targets, especially those related to small-molecule binding, as well as in discovering novel chemotypes of inhibitors through the development of new screening methods. 

## 2. Targeting β-Lactamases: Innovative Technologies and Promising Chemotypes

Among the four classes of β-lactamases, class A β-lactamases are the most frequently observed and well-studied, consisting of many clinically important β-lactamases such as TEM, SHV, CTX-M, and KPC-type enzymes [12]. In recent years, carbapenemases have caused increasing concern due to their ability to hydrolyze nearly all β-lactam antibiotics, including carbapenems [13,14]. Carbapenemase enzymes are represented by KPC-2 (class A), CMY-10 (class C), OXA-48 (class D), and NDM-1 (class B) [15,16,17,18]. While KPC-2 is the predominant carbapenemase in clinical isolates, NDM-1 and OXA-48 have also caused increasing concerns [19]. In addition, class B β-lactamases generally have broad substrate activity and include many clinically relevant carbapenemases such as VIM-2 and IMP-1 [20].

A major strategy to combat antibiotic resistance is the therapeutic combination of β-lactams with β-lactamase inhibitors (BLIs) [21]. BLIs target β-lactamases to prevent β-lactam hydrolysis, therefore keeping the antibiotic intact and capable of acting on PBPs. FDA-approved BLIs include the classical inhibitors clavulanic acid, tazobactam, and sulbactam, and the relatively new compounds avibactam and vaborbactam, all of which are designed to be used in combination with a respective β-lactam [22,23,24]. Unfortunately, resistance to β-lactam-BLI combinations has been identified both in the laboratory and clinical settings, including against avibactam and vaborbactam, even before their approval by the FDA [25,26,27]. Recent studies have demonstrated antibiotic resistance against combination therapies involving the latest BLIs and against new β-lactams, such as durlobactam [28], relebactam [29,30], zidebactam [31], tazobactam [32], taniborbactam [33], thiol-containing BLIs under development [34], and cefiderocol [35]. The resistance mechanisms include upregulation of efflux, mutations in the β-lactam target PBPs, and expression of β-lactamases and mutants less susceptible to the specific BLI, such as KPC-109 [36], NDM-9 [33], IMP-6 [34], and CMY-178 [37]. These resistance mutants highlight the need for the antimicrobial field to constantly explore novel inhibitor chemotypes to counter future resistance. 

### 2.1. Understanding the β-Lactamase Targets

β-Lactamases are among the most studied enzymes not only because of their biological importance but also because of their well-behaved properties that make them amenable to a variety of laboratory techniques. Unlike the larger and usually membrane-anchored cell wall transpeptidases, many clinically important β-lactamases are soluble and stable proteins of approximately 300–400 residues. Their relatively small size and well-behaved properties enable analysis by both X-ray crystallography and NMR [38,39,40,41,42]. Many of these enzymes yield crystals that routinely diffract to resolutions higher than 2 Å and frequently atomic (<1.2 Å) or even subatomic (<0.8 Å) resolutions, revealing hydrogen atom positions [43]. This has allowed easy characterization of the three-dimensional structures and their use in structure-based inhibitor design. In addition, both SBLs and MBLs have been subjected to time-resolved X-ray crystallographic analysis, where the catalytic reaction is tracked inside the crystal step by step [44,45,46,47]. This vast amount of structural information provides valuable resources for inhibitor discovery against these enzymes.

The active sites of the SBLs are highly similar between the three classes, all containing the S-X-X-K motif with the catalytic serine. The substrate binding pocket is well defined and relatively rigid, even though the dynamics of active site elements, especially the Ω loop in class A enzymes, can play a role in substrate binding and catalysis [9]. Ligand-induced conformational changes are usually small, except for a few cases seen in class C enzymes where the rearrangement of the R2-loop has been observed [48]. These features reduce the difficulty of modeling during structure-based inhibitor design. In comparison, the active site of MBLs is more open and contains several flexible loops. Like KPC-2 carbapenemase, MBLs such as NDM-1 contain a relatively large hydrophobic binding surface compared with other β-lactamases [49]. These features enable them to increase the binding affinity for a wide range of β-lactam substrates, but at the same time make them susceptible to small-molecule inhibition.

Recent studies concerning β-lactamases have focused on the MBL catalytic mechanism, SBL-ligand interactions such as substrate profile and inhibitor mechanism during the continuing evolution of SBL, and the impact of β-lactamase expression on the host bacteria. These experiments provide valuable insights into new inhibitor discoveries, especially those related to mechanism-based inhibitors. For β-lactam hydrolysis by MBLs, building upon previous studies [50], crystallographic analysis combined with QM/MM calculations suggests that during β-lactam ring opening, protonation of the leaving group can occur at the amide N via a metal-bound water or at C2 from a water in bulk solvent [51]. On a cellular level, the fitness cost of MBL expression in different bacteria has been found to contribute to the dissemination of these β-lactamases, which can guide the development of MBL inhibitors to treat specific bacterial infections [52]. This is reminiscent of the finding that expression of OXA and ADC SBLs in *Acinetobacter baumanni* can cause cellular defects [53,54], in contrast to the minimal fitness cost of AmpC production in *Pseudomonas aeruginosa* [55]. Interactions with other bacterial proteins can also influence MBL evolution. One study has shown that the availability of Zn(II) exerts evolutionary pressure on MBLs because less ordered conformations of nonmetalated NDM-1 can be recognized by periplasmic proteases, causing many NDM variants to contain hydrophobic mutations that induce rigidity, thereby preventing protease detection [56]. 

The studies of β-lactam hydrolysis by SBLs have focused on extended-spectrum β-lactamases (ESBLs) and carbapenemases in their interactions with extended-spectrum β-lactam antibiotics (e.g., ceftazidime) or carbapenems. The mechanism of class A SBLs is commonly studied. A recent investigation of *Mycobacterium tuberculosis* BlaC revealed an open and closed state of the active site, where the open state allows for hydrolysis of ceftazidime but without the usual contribution of E166 [57]. Analysis of CTX-M-14 ESBL demonstrated the contributions of specific residues to substrate binding and catalysis, elucidating differences between β-lactam classes. For example, Ω loop residues are required for ampicillin and cefotaxime hydrolysis but not ceftazidime [58,59]. Further insights are provided by examination of the binding of β-lactamase inhibitory protein (BLIP) to CTX-M-15, which is associated with a change in the active site 103–106 loop, thus inducing a switch in conformations, as well as controlling antibiotic hydrolysis and inhibitor susceptibility [60]. Most SBL carbapenemase studies have investigated KPC-2, revealing that the flexibility of the Ω loop allows for broad-spectrum enzymatic activity [61], and that W105 controls the transition between permissive and nonpermissive states [62]. Furthermore, it was found that N170 blocks interactions between the carbapenem hydroxyethyl group and catalytic water, and residues involved with the E166 base environment and the Q214-R220 active site loop placement are essential in the deacylation of carbapenems by KPC-2 [63,64]. Structural features involving protein flexibility of class D SBLs have also been studied [65]. Disruption in the β5–β6 loop of OXA-160 and overall active-site plasticity of OXA 24/40 allow for catalytic efficiency [66], and the positioning of specific residues like V120 and Y211 contributes to the carbapenemase activity of OXA-48 [67,68]. In addition, class D enzymes have been found to hydrolyze 1β-methyl-substituted carbapenems through β-lactone products [69]. QM/MM calculations were further employed to examine the substrate preference of OXA-48 and demonstrate that the 1β-methyl group of meropenem affects its hydrogen bonding pattern with the diacylation water, resulting in a slower hydrolysis rate compared with imipenem [70].

The binding of vaborbactam and avibactam to class A β-lactamases has been widely studied. Comparing vaborbactam binding to KPC-2 and CTX-M-14 reveals the insertion of the exocyclic oxygen into the oxyanion hole of both enzymes, resembling an acylation transition state mimic but with a more compact overall binding pose in KPC-2 [71]. In contrast, avibactam forms a covalent acyl-enzyme with SBL, adopting the chair conformation of S70 in KPC-2s shallow active site [72]. The stability of this acyl-enzyme complex appears to originate from the hindrance of proton transfer between the neutral states of E166 and K73 [73], preventing E166 from activating the catalytic water while kinetically favoring the recyclization of avibactam [74]. Compared with avibactam, desulfation of another diazabicyclooctane (DBO) inhibitor, relebactam, in KPC-2 was not observed due to the distance of active site waters from the sulfate group, indicating increased stability of the relebactam-KPC-2 complex [75]. Structural features have also been identified that contribute to resistance in clinical mutants against the ceftazidime-avibactam combination, such as P104R/V240G in KPC-4 that allows for the suitable positioning of the β-loop for ceftazidime hydrolysis [76]. It was found that a resistant D179N variant of KPC-2 contains a disruption in the salt bridge with R164 and a destabilized Ω loop, allowing for the accommodation of ceftazidime [77]. 

### 2.2. Screening Methods for β-Lactamase Inhibitors

The β-lactamase activity assay is well established, using nitrocefin or CENTA as substrates [78]. When nitrocefin is hydrolyzed, a color change can be measured at an absorption between 380 and 500 nm [79]. Umbelliferone-derived cephalosporins have also been identified as fluorogenic substrates to be used in assays for MBLs, requiring a lower enzyme concentration and offering increased sensitivity and kinetic parameters compared to traditional nitrocefin assays [80]. Additional screening methods include microscale thermophoresis (MST) with a fluorescent label and label-free surface plasmon resonance (SPR) [81,82]. Cell-based screens are yet another method of inhibitor screening against β-lactamases. For example, a collection of naturally derived products from environmental microorganisms was screened against an NDM-1-producing *Escherichia coli* strain [83]. One promising hit, aspergillomarasmine A (AMA), was obtained, and its activity was confirmed using a nitrocefin assay [84,85]. A more recent study performed cell-based screens with a DNA-encoded triazine library (DECL) against OXA-48 [86]. Compound hits from DECL screening were then synthesized without DNA tags and assessed by a nitrocefin assay. An innovative luminescence-based assay has also been developed, coupling the activation of the transcriptional factor AmpR following β-lactam exposure to the transcription of the bacterial luciferase luxCDABE operon, producing luminescence [87]. This enables inhibitor testing against a specific β-lactamase inside the cell, similar to other cell-based methods capable of screening peptidoglycan-targeting compounds [88]. 

With the advancement of computational power, virtual screening has become an increasingly popular technique in drug discovery [89]. This cost-effective and time-saving method is frequently chosen by researchers to identify non-covalent inhibitors of SBLs and MBLs, sometimes in conjunction with fragment-based approaches. Using the Specs database of drug-like compounds, hits against CTX-M-15, KPC-2, NDM-1, and VIM-2 were screened with FLAPdock, where top compounds were selected according to FLAP S-score, chemical diversity, and hydrogen bond formation, and further tested in vitro [90]. An ultra-large database screening led to the discovery of a 77 nM phenolate inhibitor of AmpC (ZINC549719643) [89]. A useful technique is to couple virtual screening experiments with NMR to test ligand binding. In one study, a fragment library for NDM-1 was created by docking [91]. Then, using saturation transfer difference (STD) NMR to measure protein-ligand interactions, mixtures of these fragments were screened in the presence of NDM-1 to identify leads. A similar approach was used against VIM-2, where virtual screening of the Vitas-M Laboratory library was performed using AutoDock Vina [92]. The top compounds were tested with ^1^H CPMG NMR for their binding to VIM-2. Interestingly, the most potent compounds inhibited VIM-2 without relying on chelation interactions.

An important aspect of inhibitor screening is the compound library. Whereas most recent efforts have focused on synthetic molecules, natural products offer a valuable source of privileged scaffolds [93,94]. Similar to AMA, the fungal metabolite, emerione A, was found to inhibit NDM-1 with an IC_50_ of 12.1 μM [95]. Fisetin, a flavonoid found in several fruits and vegetables, was found to be an inhibitor of MBLs [96]. While a related flavonol derivative, taxifolin, was also shown to inhibit VIM-2-producing *P. aeruginosa* [97]. Isolates from *Clutia myricoides*, a plant originating in the Arabian Peninsula, were found to have activity against ESBL-expressing *Klebsiella pneumoniae* strains [98]. Natural products, particularly plant extracts, have thus proven effective sources to successfully uncover β-lactamase inhibitors.

For evaluating the antibacterial activity of new antibiotics, the United States Centers for Disease Control and Prevention (CDC) and the Food and Drug Administration (FDA) have developed an Antibiotic Resistance Isolate Bank that contains a collection of resistant bacteria isolates that is free of charge to researchers [99]. The goal of the AR Isolate Bank is to guide the development of antibiotics, diagnostic tests, and assays and study pathogenic mechanisms of resistance. Additionally, the National Institutes of Health (NIH) has multiple compound collections, including the Molecular Libraries Small Molecule Repository (MLSMR), NExT diversity libraries, and other assorted libraries only found at the National Center for Advancing Translational Sciences [100]. These resources are a helpful starting point for a drug discovery campaign.

### 2.3. Novel β-Lactamase Inhibitor Scaffolds

**Figure 3:**
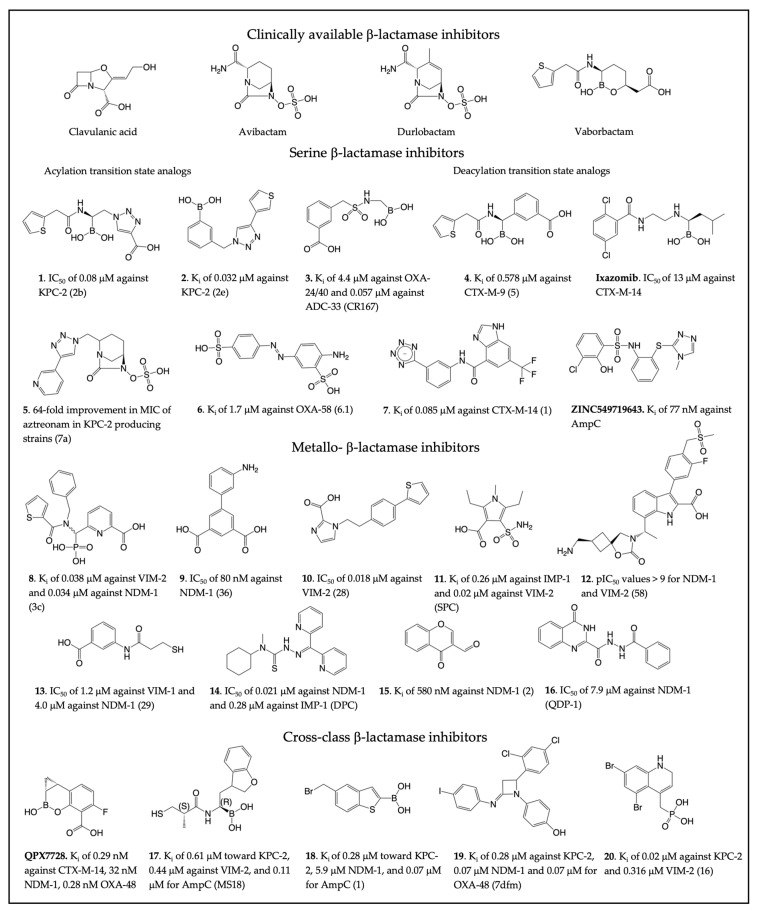
Clinically available β-lactamase inhibitors and select β-lactamase inhibitors. The compound numbers/names for the new inhibitors are shown in bold in the figure and main text. The compound name in the original publication is provided in parenthesis. (**1** [105], **2** [106], **3** [107], **4** [108], **Ixazomib** [109], **5** [110], **6** [111], **7** [112], **ZINC549719643** [89], **8** [113], **9** [114], **10** [115], **11** [116], **12** [117], **13** [118], **14** [119], **15** [120], **16** [121], **QPX7728** [122,123], **17** [124], **18** [125], **19** [126], **20** [127]).

A wide range of β-lactamase inhibitors have been developed over the years, as summarized by several recent reviews [22,101,102]. Here, we highlight some representative compounds. Whereas classical β-lactamase inhibitors, such as clavulanate, were active only against class A enzymes, the latest drug discovery efforts have focused on MBL inhibitors [103] and cross-class activity compounds [104], including those active against multiple classes of SBLs or even all classes (Figure 3). These novel inhibitor chemotypes have led to new combination therapies targeting β-lactam resistance and a better understanding of β-lactamase activity.

#### 2.3.1. Serine β-Lactamase Inhibitors

The interactions between SBL inhibitors and target enzymes mimic three states of the reaction: the non-covalent substrate/product complexes, the acyl-enzyme covalent adduct, or the acylation/deacylation TS states. The most promising new chemotypes of SBL inhibitors include boronic acid-based inhibitors and DBOs. Boronates and DBOs were also found to target PBPs [128], and they will be further discussed in the subsequent section. 

Boronic acids are known SBL inhibitors due to their ability to covalently and reversibly bind to β-lactamases in a competitive manner [129,130]. These compounds have been of particular interest as acylation transition state analogs. The boron acts as an electrophile to mimic the β-lactam carbonyl carbon, forming a tetrahedral adduct with the catalytic serine. In addition to cyclic boronates such as vaborbactam, alkyl boronic acids have been extensively studied. For example, a series of inhibitors were synthesized to mimic functional groups of certain β-lactams, and specific modifications were identified within the main scaffold that created compound **1** (Figure 3), with an IC_50_ of 0.08 μM against KPC-2 and 0.130 μM against SHV-1 [105]. Compound **2**, a phenylboronic acid inhibitor derivative acting against KPC-2 SBL, was also identified from a small compound library [106]. Compound **2** had a K_i_ of 0.032 μM and an MIC of < 0.06 μg/mL, which can act as a guide for a new class of boronic acid inhibitors. In another study, boronic acid inhibitors were developed to target class D enzymes while retaining activity toward class C [107]. The benzyl sulfonamide derivative **3** has a K_i_ of 4.4 μM against OXA-24/40 and 0.057 μM against ADC-33.

While acylation transition state analogs are more common for boronic acid inhibitors, researchers have been able to synthesize deacylation state analogs. The glycylboronic acid, compound **4**, binds to CTX-M-9 in a tetrahedral adduct mimicking the deacylation transition state, with a K_i_ of 0.578 μM against CTX-M-9 [108]. Crystallography revealed that the boronic acid oxygen replaces the catalytic water. It was found that the boron-based proteasome inhibitor **Ixazomib** also mimics the deacylation transition state of β-lactam hydrolysis [109]. Though this is a promising lead for a repurposed drug, **Ixazomib** only contained moderate inhibition towards CTX-M-14, with an IC_50_ of 13 μM. Similar boronic acid compounds have also been synthesized that display tetrahedral geometry, with interactions mimicking the deacylation intermediate and displacing the catalytic water [131,132]. 

Since their discovery in the early 2000s, the DBO class has been intensively researched and modified. Avibactam was the first DBO approved by the FDA [133,134], where the DBO amide group targets the SBL active-site serine via a carbamylation reaction [135]. Clinically available durlobactam demonstrates broadspectrum activity against SBLs, including class D OXA carbapenemases [136,137]. Substitutions in the side chain and the core rings of the DBO scaffold have been explored to form specific interactions with certain β-lactamases or increase the overall reactivity. Triazolesubstituted DBO compound **5** showed a 64-fold improvement in the MIC of aztreonam in KPC-2 and CTX-M-15-producing strains [110]. Although not as active as avibactam, it demonstrates how protein interactions involving the DBO side chain can affect the compound’s activity. 

Non-covalent compounds are of importance for β-lactamase drug discovery and functional study because they act as reversible competitive inhibitors and are also typically less toxic. Fragment screening of low-molecular-weight compounds against class D OXA-48 was performed to identify potential inhibitor scaffolds [111]. The azobenzene-based compound **6** inhibits OXA-58 with a K_i_ of 1.7 μM and OXA-48 with a K_i_ of 7.9 μM. Fragment-based virtual screening and subsequent optimization also led to the identification of the aryl tetrazole scaffold, compound **7**, that is a potent (K_i_ of 0.085 μM) non-covalent inhibitor towards CTX-M-14, proving that tetrazoles can act as a new chemotype for SBL inhibitors [112]. Interestingly, the binding of this compound desolvates the enzyme active site, altering the protonation states of Lys73 and Glu66 while promoting a short, low-barrier hydrogen bond between Lys73 and Ser70 [138]. A more recent high-affinity non-covalent β-lactamase inhibitor is the aforementioned **ZINC549719643**, identified against AmpC directly from virtual screening of the ultra-large compound library [89]. It represents one of the most active non-covalent inhibitors in vitro and demonstrates the potential of ultra-large database screening.

#### 2.3.2. Metallo-β-Lactamase Inhibitors

Clinical β-lactamase inhibitors utilize the catalytic serine of SBLs for their mode of covalent inhibition. The lack of this catalytic serine in MBLs is the main reason why many BLIs cannot target this enzyme class. Metallo-β-lactamases are evolutionarily distinct from SBLs and differ in size and topology. Several boronate-based inhibitors, such as taniborbactam, are effective against B1 MBLs and are currently in clinical trials [139,140].

Since MBLs belong to a group of metalloproteins, they can be effectively inhibited by metal chelators. In one study, a class of pyridine-2-carboxylate chelating agents were investigated, which are known inhibitors of zinc-containing enzymes [113]. The top compound (**8**), had a K_i_ of 34 nM against NDM-1. Crystallographic studies determined that the compound removed Zn2 from B3 MBLs or mimicked the interaction of β-lactam substrates in B1 active sites. A dipicolinic acid scaffold was also identified by a metal-binding pharmacophore (MBP) library targeting the Zn2 site of B1 MBLs [114]. Through a fragment-based drug design approach, 2,6-dipicolinic acid (**9**) was developed with an IC_50_ of ~80 nM against NDM-1 and was shown to form a stable NDM-1 Zn2 inhibitor ternary complex. Furthermore, a 1H-imidazole-2-carboxylic acid pharmacophore that targets Zn2 and positively charged active site residues was used to identify compound **10**, which has an IC_50_ value of 0.018 μM against VIM-2 and showed a 16-fold reduction in the MIC of meropenem [115]. This series was further developed to enhance potency by adding a 2-aminothiazole-4-carboxylic acid core [141]. In addition, screening of a small molecule library towards IMP-1 led to the identification of 2,5-dimethyl-4-sulfamoylfuran-3-carboxylic acid (SFC) but lacked significant activity towards NDM-1 and VIM-2 [116]. To improve activity, the core ring was changed to a pyrrole to yield 2,5-diethyl-1-methyl-4-sulfamoylpyrrole-3-carboxylic acid (**11**), with a K_i_ of 0.26 μM against IMP-1, 0.84 μM against NDM-1, and 0.02 μM against VIM-2. From a fluorescence-based screen of European Lead Factory (ELF) compounds against NDM-1, indole carboxylates (InCs) were recently discovered as reversible, non-covalent inhibitors that are structurally similar to carbapenems [117]. Compound **12**, was identified with pIC_50_ values of 10.2 for NDM-1 and >9.2 for VIM-2, with some InCs from the same study also showing moderate activity against SBLs.

Other studies have investigated additional metal-binding groups. A series of N-aryl mercaptopropionamide derivatives were tested against clinically relevant MBLs [118]. Thiol-based lead compound **13** displayed an IC_50_ of 4.0 μM against NDM-1 and 1.2 μM against VIM-1 and showed a synergistic effect with imipenem, reducing the MIC 256-fold against NDM-1-producing strains. Dipyridyl-substituted thiosemicarbazone (Dpc) compound **14**, a chemotherapeutic for lung cancer, was found to be a broad-spectrum inhibitor of multiple MBLs [119]. **14** had an IC_50_ of 0.021 μM against NDM-1, 0.28 μM against IMP-1, and 0.11 μM against VIM-2. Molecular docking demonstrated that the sulfur atom of thiosemicarbazone acts as the Zn binding group. 

As described earlier [92], not all MBL inhibitors depend on metal interactions for their binding affinity. Inhibitors with unique mechanisms have also been identified. Compound **15** represents a reversable covalent inhibitor for MBL, forming a covalent bond with the amine group of Lys224 within the active site of NDM-1, and has a K_i_ of 580 nM against NDM-1 [120]. Another recent HTS campaign has identified an allosteric NDM-1 inhibitor, **16** [121], echoing other studies of allosteric regulation of SBL enzymatic activity [142,143]. 

#### 2.3.3. Dual Action β-Lactamase Inhibitors

Bacteria are capable of producing multiple types of β-lactamases, exacerbating the clinical concern of antibiotic resistance. The aim of developing dual action inhibitors is to target both SBLs and MBLs, but the evolutionary, structural, and mechanistic differences between the two types of enzymes pose difficulties in designing dual inhibitors [104]. Boronates are a promising lead scaffold for cross-class inhibition since they mimic the tetrahedral intermediate of β-lactamase substrates (Figure 2). Taniborbactam, from Venatorx, is a potent cross-class inhibitor with an IC_50_ of 0.03 μM against KPC-2, 0.02 μM against VIM-2, and 0.42 μM against OXA-48 [144]. Another cyclic boronic acid, **QPX7728** from Qpex Biopharma, displays potent activity against SBLs and MBLs from all four classes, with a K_i_ of 0.29 nM against CTX-M-14, 32 nM against NDM-1, 8.5 nM against AmpC, and 0.28 nM against OXA-48 [122,123]. In combination with several β-lactams, **QPX7728** has demonstrated broad-spectrum antibacterial activity, including against strains resistant to other β-lactam-BLI combinations. 

In an attempt to develop dual-action inhibitors, researchers designed compounds containing pharmacophores found in both SBL and MBL inhibitors, identifying **17** with an α-aminoboronic acid and captopril motif [124]. In MBLs, **17** was a metal chelator, and in SBLs, a covalent adduct was formed in KPC-2 between Ser70 and the boronic acid. **17** displayed a K_i_ of 0.61 μM toward KPC-2, 0.44 μM against VIM-2, and 0.11 μM for AmpC. Other noncyclic boronic acids were also found to be active against four classes of β-lactamases. Starting from benzo[b]thiopene-2-boronic acid derivatives, researchers identified compound **18**, with K_i_ values ranging from 2.8 μM towards KPC-2, 5.9 μM towards NDM-1, and 0.07 μM against AmpC [125,145]. 

Other unique dual action scaffolds include imino-analogues of β-lactams, where various aryl groups were substituted around the azetidinimine scaffold, resulting in a phenol compound, **19**, with a K_i_ of 0.28 μM against KPC-2, 0.07 μM against NDM-1, and 0.07 μM for OXA-48 [126]. Additionally, a non-covalent heteroaryl phosphonate scaffold was discovered through molecular docking, leading to compound **20**, which inhibits KPC-2 with a K_i_ of 0.020 μM and 0.316 μM against VIM-2 [127]. 

## 3. Targeting Transpeptidases: Old Challenges and New Opportunities

**Figure 4:**
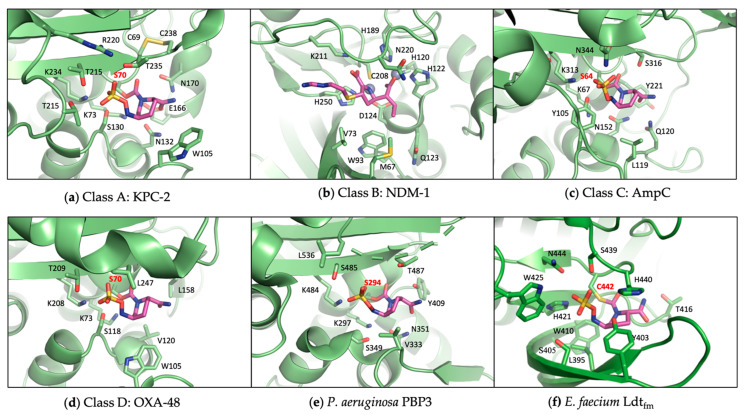
Active sites of representative β-lactamases and transpeptidases. The catalytic serine and cysteine are labeled in red, highlighting the similarities between SBLs and PBPs. (**a**) Class A SBL KPC-2 in complex with avibactam (PDB 4ZBE); (**b**) Class B MBL NDM-1 in complex with imipenem (PDB 5YPL); (**c**) Class C SBL AmpC in complex with avibactam (PDB 6LC8); (**d**) Class D SBL OXA-48 in complex with avibactam (PDB 4WMC); (**e**) *P. aeruginosa* PBP3 in complex with avibactam (PDB 7KIV); (**f**) *Enterococcus faecium* Ldt_fm_ in complex with avibactam (PDB 6FJ1).

Serine β-lactamases evolved from PBPs, turning a suicide substrate back into a real substrate [2,146]. Due to the evolutionary and functional relationship, SBLs, PBPs, and Ldts share a number of active site features for ligand binding and interact similarly with many SBL inhibitors (Figure 4). For example, avibactam and other DBO compounds have been found to inhibit PBPs and Ldts to various degrees [128,147,148,149,150]. Boronic acid compounds have also been shown to react with the catalytic serine of PBPs, like with other peptidases [151,152,153]. The development of new BLIs thus offers new opportunities for novel antibiotic discovery against cell wall transpeptidases. 

### 3.1. Understanding the Transpeptidase Targets

Although β-lactams have been known to target PBPs for a long time, our knowledge of these proteins, and to an even greater extent of Ldts, remains limited. This is partly due to the remarkable broad-spectrum efficacy of β-lactams, which may have reduced the perceived value of these proteins as targets for novel antibiotic development. One of the exceptions is the investigation of PBPs and Ldts and their roles in β-lactam resistance. One PBP receiving particular attention was methicillin-resistant *Staphylococcus aureus* (MRSA) PBP2, shown to be able to discriminate against β-lactam binding in favor of the peptide substrate using an allosteric mechanism [154,155,156,157,158,159]. Other β-lactam-insensitive PBPs include PBP4 from *Enterococcus faecalis* and PBP5 from *Enterococcus faecium* [160,161]. Similarly, PBP 2x and PBP 2b, essential monofunctional transpeptidases during septal and peripheral peptidoglycan synthesis in different strains of *Streptococcus pneumoniae* [162,163], have been shown to confer antibiotic resistance through mutations [162,164]. In addition, a recent study has suggested that *Clostridioides difficile* PBP2 is less susceptible to cephalosporins than other β-lactams [165]. Furthermore, some PBPs have been shown to exhibit significant β-lactamase activity and may contribute to β-lactam resistance [166,167]. Aside from PBPs, Ldts also play an important role in β-lactam resistance. This is represented by *M. tuberculosis* Ldt2, which is insensitive to most β-lactams except carbapenems and has been extensively studied in new antibiotic development [4,168,169].

Compared with the vast amount of structural information concerning β-lactamases, the structures of most PBPs and Ldts remain unknown, including for many clinically important bacterial pathogens. Whereas many β-lactamases are plasmid-borne and exhibit few variations among different bacteria, the peptidoglycan transpeptidases from each bacterial species may have unique features. Most recently, two groups reported the identification of highly conserved zinc-binding domains in *C. difficile* and *A. baumannii* PBP2, which in *C. difficile* was found to be an essential transpeptidase for cell growth [165,170]. This motif in PBP has never been observed in any other known PBP structures, but sequence analysis has suggested its prevalence in many bacteria, particularly the *Firmicutes* [165]. The specific function of this motif in bacteria remains to be determined, demonstrating that our knowledge of these enzymes can still be expanded. 

As each bacterium has multiple PBPs and Ldts, one key challenge in rational drug design against these enzymes is to determine which ones represent the best antibiotic target and how polypharmacology may enhance antibiotic efficacy by inhibiting multiple proteins. For many bacteria, the value of each PBP for drug discovery is only starting to be unraveled. For example, some studies have identified PBP3 as an essential transpeptidase for *P. aeruginosa* growth and therefore a key β-lactam target (Figure 4) [171,172,173], while PBP2 may also represent a good therapeutic target for novel antibiotic discovery [128]. Other researchers used *Bacillus subtilis* cells to visualize and study PBP inhibition profiles of β-lactam antibiotics in live cells, which allowed the determination of PBPs essential for the growth of the microorganism and their roles in antibiotic resistance [174]. The advancement of artificial intelligence has enabled the combination of a medium-throughput image-based assay with machine learning to automatically analyze the activity and polyspecificity of β-lactams against *E. coli* cells [175]. Related PBP-occupancy experiments have been carried out for various β-lactams in other bacteria [176,177]. Novel assays have also been developed to simultaneously measure the outer membrane permeability of various β-lactams against carbapenem-resistant *K. pneumoniae*, *Enterobacter cloacae*, *E. coli*, and *P. aeruginosa* [178,179,180,181], which offers important insights into the interactions between β-lactams and PBPs in situ. Furthermore, there has been increasing interest in improving treatment efficacy by targeting multiple PBPs and potentially some β-lactamases as well, by combining several β-lactams [182,183]. In other cases, researchers used machine learning to optimize antibiotic combinations of β-lactams and other antibiotics, such as meropenem and polymyxin B [184]. To probe the targets and mechanisms of the actions of β-lactams and β-lactamase inhibitors, a chemo-genetic approach was also recently employed by constructing a transposon mutant library of *Burkholderia cenocepacia* and evaluating the mutant fitness after exposure to cell-envelope-targeting antibiotics [185]. The studies offered valuable insights into the cellular activities of avibactam, cefedericol, and other antibiotics, while also providing a useful strategy to identify antibiotic targets and explore antibiotic combinations. 

### 3.2. Screening Methods for Transpeptidase Inhibitors

The lack of efficient biochemical techniques to evaluate transpeptidase activity and inhibition is one of the challenges facing the development of novel antibiotics. The most widely employed method to assess the activity of antibiotics towards PBPs is a competition assay using BOCILLIN FL, a fluorescent penicillin that binds to PBPs [186]. PBPs bound by BOCILLIN FL can be visualized by gel electrophoresis. A novel and easier fluorescence anisotropy assay has also been developed to measure the acylation rate of PBPs in the absence or presence of inhibitors [187], although it is not applicable to all PBPs, even if they react with BOCILLIN FL. Other similar probes include fluorescent carbapenems. Some of these compounds have been developed for assaying carbapenemases [188,189], while others can be useful for detecting and labeling PBPs and Ldts [190,191].

Another method used to determine transpeptidase activity for PBPs is to measure the enzyme’s ability to hydrolyze an analog of the bacterial cell wall stem peptides, usually a thioester of hippuric acid [152,192,193,194,195]. The released mercaptoacetate product can then be quantified by colorimetric or fluorescent dyes. Ultra-performance liquid chromatography and mass spectrometry (UPLC-MS) is another powerful analytical technique that provides scientists with specific information about transpeptidase acylation by antibiotics. Coupling mass spectrometry with liquid chromatography allows for the separation of reaction components and the accurate mass determination of products. This method has been applied by numerous scientists to monitor the acylation rate of carbapenems by *M. tuberculosis* _L,D_-transpeptidase [4,196,197,198]. Similar experiments have been performed on PBPs to analyze the crosslinking products using Lipid-II substrates labeled with a biotinylated probe (biotin-D-Lys) and isolated directly from bacterial cells [199].

Computational methods have been a highly effective tool for BLI discovery. Similarly, numerous in silico studies have been initiated to target PBPs in an attempt to tackle the urgent threat of antimicrobial resistance. To identify promising lead compounds, large libraries of commercially available small molecules are used to perform docking-based virtual screening [200,201]. Additional computational methods such as structure-activity relationship modeling, molecular dynamics simulations, and pharmacophore modeling are applied to selected compounds with high predicted affinity towards drug targets [202,203,204,205,206,207], even though the results from some of these computational efforts have yet to be experimentally validated. Even when more traditional methods were used to identify potential antimicrobial hits, such as cell-based screening of fungal metabolites or high-throughput screening (HTS), the integration of computational methods like inverse molecular docking proved instrumental. Indeed, docking allows the determination of a metabolite’s target, the underlying mechanism of inhibition, and the binding pose adopted by the compound in the active site of its target [208,209]. 

Experimental HTS has also been extensively applied to identify PBP inhibitors. In one study, 30,000 compounds were screened against the MRSA USA3000 strain to identify small-molecule inhibitors of *S. aureus* PBP4 [210]. Three compounds were identified to modulate PBP4 indirectly by limiting protein transcription, while two other compounds did not impact PBP4 transcription and therefore were believed to affect the protein’s function. Another study implemented high-throughput crystallography to explore the interactions of a potent SBL inhibitor chemotype with *P. aeruginosa* PBP3 [152]. Protein crystals were soaked with boron-containing fragments to obtain inhibitor-protein complexes. Crystal structures revealed a covalent linkage between the boronic acid compounds and PBP3, leading to the discovery of a novel scaffold as a potential PBP inhibitor.

### 3.3. New Transpeptidase Inhibitor Scaffolds

The drug discovery efforts against bacterial cell wall transpeptidases have used multiple strategies, including: optimization of conventional β-lactams to enhance membrane permeability or resistance to β-lactamase degradation; derivatizing non-β-lactam scaffolds such as γ-lactam, boronic acid, DBO, and β-lactone; and exploration of additional novel chemotypes through in silico or experimental screening. Most of these experiments focus on PBPs, while new carbapenems have been developed specifically against Ldts. 

**Figure 5:**
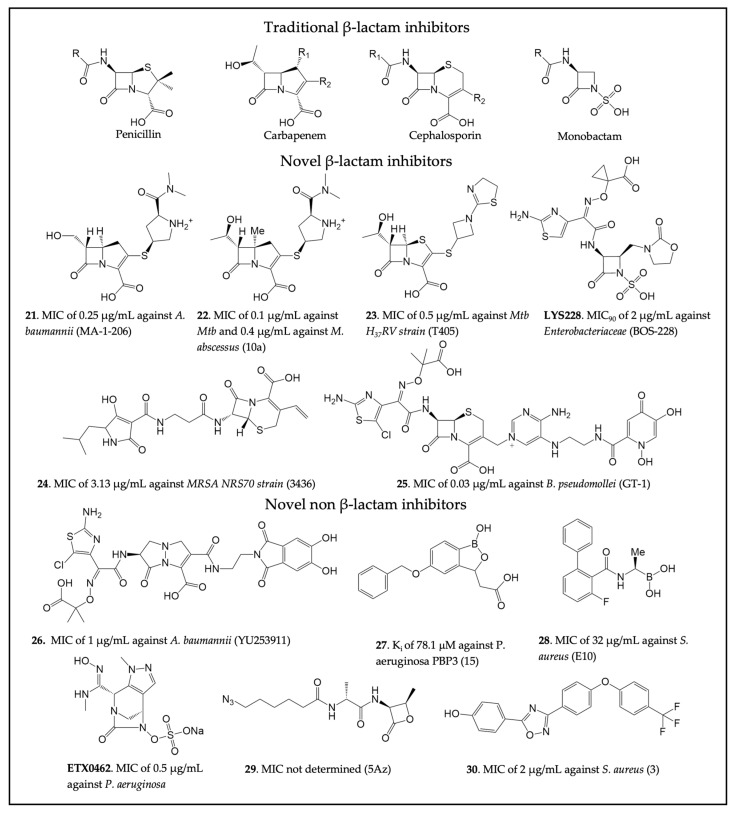
Four classes of β-lactam antibiotics and recently developed novel cell wall transpeptidase inhibitors. The compound name in the original publication is provided in parenthesis. (**21** [211], **22** [196], **23** [212], **LYS228** [213], **24** [217], **25** [218], **26** [219], **27** [152], **28** [220], **ETX0462** [221], **29** [222], **30** [223]).

For new β-lactams, many studies have been carried out to improve the activity spectrum of carbapenems. For instance, building upon the traditional carbapenem scaffold, the C6 hydroxyethyl group was replaced with a hydroxymethyl to prevent access to the deacylating water and thus inhibit OXA-23 [211]. This resulted in **21** (Figure 5), which is 8-fold more potent than meropenem towards *A. baumannii* and achieves OXA-23 inhibition by preventing deacylation through the formation of a hydrogen bond between the C6 hydroxymethyl and Lys82. Similarly, a C5α-methyl substitution of the carbapenem scaffold led to compound **22** and displayed a 10-fold improved potency compared to meropenem against *M. tuberculosis* [196]. The C5α-methyl carbapenem showed decreased hydrolysis by BlaC but acylation rates of Ldt_Mt2_ comparable to meropenem. Likewise, penem modifications to the C2 side chain led to the identification of **23** as a potent antimicrobial against Ldt_Mt2_ of *M. tuberculosis* [212]. Compound **23** was shown to have an MIC of 0.5 µg/mL against the Mtb H_37_Rv strain. In exploring new avenues other than carbapenems or penems, a novel monobactam called **LYS228** (also known as BOS-228) has been developed, which showed promising activity against carbapenem-resistant *Enterobacteriaceae* [213,214]. Other studies identified additional monobactams showing promising activity against Gram-negative bacteria by targeting their PBPs [215,216]. The construction of β-lactam-tetramic acid hybrids is yet another example of the efforts made to optimize conventional β-lactam antibiotics [217]. Among these compounds, **24** showed potent activity against the multi-drug-resistant (MDR) *S. aureus* NRS70 strain (MIC values of 3.13 µg/mL).

Exploring siderophore-β-lactam conjugates led to the development of a novel siderophore cephalosporin (**25**) that was demonstrated to be effective against ESKAPE pathogens [218]. Compound **25** consists of a dihydroxypyridone siderophore conjugated to a modified aminothiazoylglycyl cephalosporin. This modification allows the drug to be more resistant to hydrolysis by ESBLs and carbapenemases. The activity of **25** was also investigated in combination with GT-055, a novel β-lactamase inhibitor, against MDR bacteria such as *E. coli*, *S. aureus*, and *P. aeruginosa*. Additionally, **25** showed good activity against other biothreat bacterial pathogens, such as *Burkholderia pseudomallei*, in vitro and in vivo, with an MIC value of 0.03 µg/mL. Similar to **25**, a γ-lactam siderophore antibiotic (**26**) effective against MDR *P. aeruginosa*, *K. pneumoniae*, and *Acinetobacter* spp. was developed [219]. The preclinical evaluation showed that compound **26** targeted *P. aeruginosa* PBP3 and had enhanced MIC_50_ values (0.5 µg/mL vs. 198 *Acinetobacter* ssp. and 1 µg/mL vs. 98 CRAB strains) when compared to meropenem.

In addition to γ-lactam as a new non-β-lactam chemotype, the structural similarities between SBLs and PBPs were exploited to study the potential of boron-containing fragments and vaborbactam against *P. aeruginosa* PBP3 [151]. Vaborbactam was shown to inhibit PBP3 with an IC_50_ value of 262 µM. Despite not being particularly potent, the inhibition suggested the possibility of exploiting the boronic acid scaffold to develop novel PBP inhibitors able to evade resistance mechanisms. As a result, a boron-containing fragment library was developed and analyzed to identify novel PBP inhibitors against *P. aeruginosa* [152]. The study generated moderately potent PBP3 inhibitors, with compound **27** shown to target key PBP active site residues, but no antibacterial activity was reported. Crystal structures of *S. pneumoniae* PBP1 in complex with different boronic acid derivatives allowed for the identification of **28**, an alkyl boronic acid compound [220]. In vivo, the compound was shown to target essential PBPs, displaying moderate antibacterial activity with an MIC value of 32 μg/mL against MRSA. Additionally, DBO and related scaffolds have also been explored to develop PBP inhibitors against Gram-negative pathogens. This effort has led to extensive optimization of the DBO structure to identify WCK 5153 [128,150]. The binding of the compound to *P. aeruginosa* PBP2 was confirmed via x-ray crystallography. However, the compound showed relatively limited antimicrobial activity by itself. A recent study identified another DBO derivative and PBP inhibitor, **ETX0462**, which represents the most active and broad-spectrum DBO compound, both in vitro and in vivo, against *P. aeruginosa* (MIC 0.5 µg/ML) and other bacteria [221]. This compound demonstrates the immense potential of DBO compounds as a new class of antibiotics. 

β-Lactones represent another new class of potential PBP inhibitors due to the chemical similarity between β-lactones and β-lactams [224]. These compounds have been used as chemical probes to selectively target specific PBPs in *S. pneumoniae* [225,226]. A successful example was compound **29**, which was shown to bind to PBP2a in silico [222]. Further insights into interactions between β-lactones and PBP1b were provided by crystallography and additional molecular docking studies. Novel PBP inhibitors with a non-β-lactam scaffold have also been identified through virtual screening against MRSA PBP2a [223]. This new oxadiazole-derived chemotype was shown to have bactericidal activity against several Gram-positive bacteria. Among the best compounds derived from this study was **30**, which was shown to have an MIC of 2 μg/mL against different antibiotic-resistant strains of *S. aureus*. Investigating the mode of action of this compound revealed that it inhibits peptidoglycan synthesis. Compound **30** was also shown to inhibit MRSA PBP2a with an IC_50_ of 8 μg/mL. Further development of this lead led to the development of a narrow-spectrum antibacterial effective against 101 strains of *C. difficile* with an MIC of 0.5 µg/mL [227]. 

## 4. Conclusions

In summary, both cell wall transpeptidases and β-lactamases represent valuable antibiotic targets and model systems for studying enzyme function and inhibition. The past decade has seen exciting progress in drug discovery against both groups of enzymes. In particular, we now have several new β-lactamase inhibitor scaffolds (e.g., DBOs, cyclic boronates) that are clinically effective or under further development, including the cyclic boronate ultrabroad-spectrum inhibitor active against all classes of β-lactamases. Some of these β-lactamase inhibitors, especially DBOs, also provide new chemical matter for novel antibiotic development against transpeptidases. Such efforts are being facilitated by new knowledge of the transpeptidase enzymes concerning their structure and in vitro and in situ interactions with β-lactams, as well as by advancements in screening technologies. Important knowledge gaps remain, as the cell wall transpeptidases from each bacterium may have unique features. Meanwhile, in comparison to many plasmid-borne β-lactamases that often make broad-spectrum inhibition desirable, most cell wall transpeptidases are chromosome-encoded, and inhibitors targeting one essential cell wall transpeptidase can have immense clinical potential. The cell wall transpeptidases will therefore offer a multitude of opportunities for future drug discovery in our efforts to understand the function of these proteins, develop new methodologies, and uncover novel inhibitors. 

## Data Availability

Not applicable.

## References

[1] Bush K., Bradford P.A. (2016). β-Lactams and β-Lactamase Inhibitors: An Overview. Cold Spring Harb. Perspect. Med..

[2] Mora-Ochomogo M., Lohans C.T. (2021). β-Lactam antibiotic targets and resistance mechanisms: From covalent inhibitors to substrates. RSC Med. Chem..

[3] Cochrane S.A., Lohans C.T. (2020). Breaking down the cell wall: Strategies for antibiotic discovery targeting bacterial transpeptidases. Eur. J. Med. Chem..

[4] Kumar P., Kaushik A., Lloyd E.P., Li S.G., Mattoo R., Ammerman N.C., Bell D.T., Perryman A.L., Zandi T.A., Ekins S. (2017). Non-classical transpeptidases yield insight into new antibacterials. Nat. Chem. Biol..

[5] Aliashkevich A., Cava F. (2022). LD-transpeptidases: The great unknown among the peptidoglycan cross-linkers. FEBS J..

[6] Darby E.M., Trampari E., Siasat P., Gaya M.S., Alav I., Webber M.A., Blair J.M.A. (2022). Molecular mechanisms of antibiotic resistance revisited. Nat. Rev. Microbiol..

[7] Blair J.M., Webber M.A., Baylay A.J., Ogbolu D.O., Piddock L.J. (2015). Molecular mechanisms of antibiotic resistance. Nat. Rev. Microbiol..

[8] Bassetti M., Garau J. (2021). Current and future perspectives in the treatment of multidrug-resistant Gram-negative infections. J. Antimicrob. Chemother..

[9] Tooke C.L., Hinchliffe P., Bragginton E.C., Colenso C.K., Hirvonen V.H.A., Takebayashi Y., Spencer J. (2019). β-Lactamases and β-Lactamase Inhibitors in the 21st Century. J. Mol. Biol..

[10] Bush K., Jacoby G.A. (2010). Updated functional classification of β-lactamases. Antimicrob. Agents Chemother..

[11] Palzkill T. (2013). Metallo-β-lactamase structure and function. Ann. N. Y. Acad. Sci..

[12] Bush K. (2018). Past and Present Perspectives on β-Lactamases. Antimicrob. Agents Chemother..

[13] Walsh T.R. (2010). Emerging carbapenemases: A global perspective. Int. J. Antimicrob. Agents.

[14] Aurilio C., Sansone P., Barbarisi M., Pota V., Giaccari L.G., Coppolino F., Barbarisi A., Passavanti M.B., Pace M.C. (2022). Mechanisms of Action of Carbapenem Resistance. Antibiotics.

[15] Papp-Wallace K.M., Bethel C.R., Distler A.M., Kasuboski C., Taracila M., Bonomo R.A. (2010). Inhibitor resistance in the KPC-2 β-lactamase, a preeminent property of this class A β-lactamase. Antimicrob. Agents Chemother..

[16] Philippon A., Arlet G., Labia R., Iorga B.I. (2022). Class C β-Lactamases: Molecular Characteristics. Clin. Microbiol. Rev..

[17] Boyd S.E., Holmes A., Peck R., Livermore D.M., Hope W. (2022). OXA-48-Like β-Lactamases: Global Epidemiology, Treatment Options, and Development Pipeline. Antimicrob. Agents Chemother..

[18] Linciano P., Cendron L., Gianquinto E., Spyrakis F., Tondi D. (2019). Ten Years with New Delhi Metallo-β-lactamase-1 (NDM-1): From Structural Insights to Inhibitor Design. ACS Infect. Dis..

[19] Kedisaletse M., Phumuzile D., Angela D., Andrew W., Mae N.F. (2023). Epidemiology, risk factors, and clinical outcomes of carbapenem resistant Enterobacterales in Africa: A systematic review. J. Glob. Antimicrob. Resist..

[20] Bush K., Bradford P.A. (2019). Interplay between β-lactamases and new β-lactamase inhibitors. Nat. Rev. Microbiol..

[21] Papp-Wallace K.M. (2019). The latest advances in β-lactam/β-lactamase inhibitor combinations for the treatment of Gram-negative bacterial infections. Expert. Opin. Pharmacother..

[22] Tehrani K., Martin N.I. (2018). β-lactam/β-lactamase inhibitor combinations: An update. Medchemcomm.

[23] Iqbal Z., Sun J., Yang H., Ji J., He L., Zhai L., Ji J., Zhou P., Tang D., Mu Y. (2022). Recent Developments to Cope the Antibacterial Resistance via β-Lactamase Inhibition. Molecules.

[24] Lang P.A., Raj R., Tumber A., Lohans C.T., Rabe P., Robinson C.V., Brem J., Schofield C.J. (2022). Studies on enmetazobactam clarify mechanisms of widely used β-lactamase inhibitors. Proc. Natl. Acad. Sci. USA.

[25] Papp-Wallace K.M., Winkler M.L., Taracila M.A., Bonomo R.A. (2015). Variants of β-lactamase KPC-2 that are resistant to inhibition by avibactam. Antimicrob. Agents Chemother..

[26] Lomovskaya O., Sun D., Rubio-Aparicio D., Nelson K., Tsivkovski R., Griffith D.C., Dudley M.N. (2017). Vaborbactam: Spectrum of β-Lactamase Inhibition and Impact of Resistance Mechanisms on Activity in Enterobacteriaceae. Antimicrob. Agents Chemother..

[27] Nichols W.W., Lahiri S.D., Bradford P.A., Stone G.G. (2023). The primary pharmacology of ceftazidime/avibactam: Resistance in vitro. J. Antimicrob. Chemother..

[28] Moussa S.H., Shapiro A.B., McLeod S.M., Iyer R., Carter N.M., Tsai Y.K., Siu L.K., Miller A.A. (2023). Molecular drivers of resistance to sulbactam-durlobactam in contemporary clinical isolates of Acinetobacter baumannii. Antimicrob. Agents Chemother..

[29] Alonso-Garcia I., Vazquez-Ucha J.C., Lasarte-Monterrubio C., Gonzalez-Mayo E., Lada-Salvador P., Vela-Fernandez R., Aja-Macaya P., Guijarro-Sanchez P., Rumbo-Feal S., Muino-Andrade M. (2023). Simultaneous and divergent evolution of resistance to cephalosporin/β-lactamase inhibitor combinations and imipenem/relebactam following ceftazidime/avibactam treatment of MDR Pseudomonas aeruginosa infections. J. Antimicrob. Chemother..

[30] Gato E., Guijarro-Sanchez P., Alonso-Garcia I., Pedraza-Merino R., Conde A., Lence E., Rumbo-Feal S., Pena-Escolano A., Lasarte-Monterrubio C., Blanco-Martin T. (2023). In vitro development of imipenem/relebactam resistance in KPC-producing Klebsiella pneumoniae involves multiple mutations including OmpK36 disruption and KPC modification. Int. J. Antimicrob. Agents.

[31] Pan X., Zhao X., Song Y., Ren H., Tian Z., Liang Q., Jin Y., Bai F., Cheng Z., Feng J. (2022). Molecular Characterization of WCK 5222 (Cefepime/Zidebactam)-Resistant Mutants Developed from a Carbapenem-Resistant Pseudomonas aeruginosa Clinical Isolate. Microbiol. Spectr..

[32] Gomis-Font M.A., Pitart C., Del Barrio-Tofino E., Zboromyrska Y., Cortes-Lara S., Mulet X., Marco F., Vila J., Lopez-Causape C., Oliver A. (2021). Emergence of Resistance to Novel Cephalosporin-β-Lactamase Inhibitor Combinations through the Modification of the Pseudomonas aeruginosa MexCD-OprJ Efflux Pump. Antimicrob. Agents Chemother..

[33] Drusin S.I., Le Terrier C., Poirel L., Bonomo R.A., Vila A.J., Moreno D.M. (2023). Structural basis of metallo-β-lactamase resistance to taniborbactam. Antimicrob. Agents Chemother..

[34] Yamaguchi Y., Kato K., Ichimaru Y., Uenosono Y., Tawara S., Ito R., Matsuse N., Wachino J.I., Toma-Fukai S., Jin W. (2023). Difference in the Inhibitory Effect of Thiol Compounds and Demetallation Rates from the Zn(II) Active Site of Metallo-β-lactamases (IMP-1 and IMP-6) Associated with a Single Amino Acid Substitution. ACS Infect. Dis..

[35] Liu X., Lei T., Yang Y., Zhang L., Liu H., Leptihn S., Yu Y., Hua X. (2022). Structural Basis of PER-1-Mediated Cefiderocol Resistance and Synergistic Inhibition of PER-1 by Cefiderocol in Combination with Avibactam or Durlobactam in Acinetobacter baumannii. Antimicrob. Agents Chemother..

[36] Pilato V.D., Codda G., Niccolai C., Willison E., Wong J.L.C., Coppo E., Frankel G., Marchese A., Rossolini G.M. (2023). Functional features of KPC-109, a novel 270-loop KPC-3 mutant mediating resistance to avibactam-based β-lactamase inhibitor combinations and cefiderocol. Int. J. Antimicrob. Agents.

[37] Zhou J., Wang W., Liang M., Yu Q., Cai S., Lei T., Jiang Y., Du X., Zhou Z., Yu Y. (2023). A Novel CMY Variant Confers Transferable High-Level Resistance to Ceftazidime-Avibactam in Multidrug-Resistant *Escherichia coli*. Microbiol. Spectr..

[38] Philippon A., Slama P., Deny P., Labia R. (2016). A Structure-Based Classification of Class A β-Lactamases, a Broadly Diverse Family of Enzymes. Clin. Microbiol. Rev..

[39] Rossi M.A., Palzkill T., Almeida F.C.L., Vila A.J. (2022). Slow Protein Dynamics Elicits New Enzymatic Functions by Means of Epistatic Interactions. Mol. Biol. Evol..

[40] Cheng K., Wu Q., Yao C., Chai Z., Jiang L., Liu M., Li C. (2023). Distinct Inhibition Modes of New Delhi Metallo-β-lactamase-1 Revealed by NMR Spectroscopy. JACS Au.

[41] Sakhrani V.V., Ghosh R.K., Hilario E., Weiss K.L., Coates L., Mueller L.J. (2021). Toho-1 β-lactamase: Backbone chemical shift assignments and changes in dynamics upon binding with avibactam. J. Biomol. NMR.

[42] Elings W., Chikunova A., van Zanten D.B., Drenth R., Ahmad M.U.D., Blok A.J., Timmer M., Perrakis A., Ubbink M. (2021). Two β-Lactamase Variants with Reduced Clavulanic Acid Inhibition Display Different Millisecond Dynamics. Antimicrob. Agents Chemother..

[43] Chen Y., Bonnet R., Shoichet B.K. (2007). The acylation mechanism of CTX-M β-lactamase at 0.88 a resolution. J. Am. Chem. Soc..

[44] Olmos J.L., Pandey S., Martin-Garcia J.M., Calvey G., Katz A., Knoska J., Kupitz C., Hunter M.S., Liang M., Oberthuer D. (2018). Enzyme intermediates captured “on the fly” by mix-and-inject serial crystallography. BMC Biol..

[45] Wilamowski M., Sherrell D.A., Kim Y., Lavens A., Henning R.W., Lazarski K., Shigemoto A., Endres M., Maltseva N., Babnigg G. (2022). Time-resolved β-lactam cleavage by L1 metallo-β-lactamase. Nat. Commun..

[46] Stewart N.K., Toth M., Stasyuk A., Vakulenko S.B., Smith C.A. (2021). In Crystallo Time-Resolved Interaction of the Clostridioides difficile CDD-1 enzyme with Avibactam Provides New Insights into the Catalytic Mechanism of Class D β-lactamases. ACS Infect. Dis..

[47] Malla T.N., Zielinski K., Aldama L., Bajt S., Feliz D., Hayes B., Hunter M., Kupitz C., Lisova S., Knoska J. (2023). Heterogeneity in M. tuberculosis β-lactamase inhibition by Sulbactam. Nat. Commun..

[48] Bhattacharya M., Toth M., Antunes N.T., Smith C.A., Vakulenko S.B. (2014). Structure of the extended-spectrum class C β-lactamase ADC-1 from Acinetobacter baumannii. Acta Crystallogr. D Biol. Crystallogr..

[49] King D., Strynadka N. (2011). Crystal structure of New Delhi metallo-β-lactamase reveals molecular basis for antibiotic resistance. Protein Sci..

[50] Park H., Brothers E.N., Merz K.M. (2005). Hybrid QM/MM and DFT investigations of the catalytic mechanism and inhibition of the dinuclear zinc metallo-β-lactamase CcrA from Bacteroides fragilis. J. Am. Chem. Soc..

[51] Lisa M.N., Palacios A.R., Aitha M., Gonzalez M.M., Moreno D.M., Crowder M.W., Bonomo R.A., Spencer J., Tierney D.L., Llarrull L.I. (2017). A general reaction mechanism for carbapenem hydrolysis by mononuclear and binuclear metallo-β-lactamases. Nat. Commun..

[52] Lopez C., Ayala J.A., Bonomo R.A., Gonzalez L.J., Vila A.J. (2019). Protein determinants of dissemination and host specificity of metallo-β-lactamases. Nat. Commun..

[53] Colquhoun J.M., Farokhyfar M., Hutcheson A.R., Anderson A., Bethel C.R., Bonomo R.A., Clarke A.J., Rather P.N. (2021). OXA-23 β-Lactamase Overexpression in Acinetobacter baumannii Drives Physiological Changes Resulting in New Genetic Vulnerabilities. mBio.

[54] Colquhoun J.M., Farokhyfar M., Anderson A.C., Bethel C.R., Bonomo R.A., Clarke A.J., Rather P.N. (2023). Collateral Changes in Cell Physiology Associated with ADC-7 β-Lactamase Expression in Acinetobacter baumannii. Microbiol. Spectr..

[55] Barcelo I.M., Jordana-Lluch E., Escobar-Salom M., Torrens G., Fraile-Ribot P.A., Cabot G., Mulet X., Zamorano L., Juan C., Oliver A. (2022). Role of Enzymatic Activity in the Biological Cost Associated with the Production of AmpC β-Lactamases in Pseudomonas aeruginosa. Microbiol. Spectr..

[56] Gonzalez L.J., Bahr G., Gonzalez M.M., Bonomo R.A., Vila A.J. (2023). In-cell kinetic stability is an essential trait in metallo-β-lactamase evolution. Nat. Chem. Biol..

[57] Sun J., Chikunova A., Boyle A.L., Voskamp P., Timmer M., Ubbink M. (2023). Enhanced activity against a third-generation cephalosporin by destabilization of the active site of a class A β-lactamase. Int. J. Biol. Macromol..

[58] Lu S., Montoya M., Hu L., Neetu N., Sankaran B., Prasad B.V.V., Palzkill T. (2023). Mutagenesis and structural analysis reveal the CTX-M β-lactamase active site is optimized for cephalosporin catalysis and drug resistance. J. Biol. Chem..

[59] Judge A., Hu L., Sankaran B., Van Riper J., Venkataram Prasad B.V., Palzkill T. (2023). Mapping the determinants of catalysis and substrate specificity of the antibiotic resistance enzyme CTX-M β-lactamase. Commun. Biol..

[60] Lu S., Hu L., Lin H., Judge A., Rivera P., Palaniappan M., Sankaran B., Wang J., Prasad B.V.V., Palzkill T. (2022). An active site loop toggles between conformations to control antibiotic hydrolysis and inhibition potency for CTX-M β-lactamase drug-resistance enzymes. Nat. Commun..

[61] Tooke C.L., Hinchliffe P., Beer M., Zinovjev K., Colenso C.K., Schofield C.J., Mulholland A.J., Spencer J. (2023). Tautomer-Specific Deacylation and Omega-Loop Flexibility Explain the Carbapenem-Hydrolyzing Broad-Spectrum Activity of the KPC-2 β-Lactamase. J. Am. Chem. Soc..

[62] Cortina G.A., Hays J.M., Kasson P.M. (2018). Conformational Intermediate That Controls KPC-2 Catalysis and Β-Lactam Drug Resistance. ACS Catal..

[63] Mehta S.C., Furey I.M., Pemberton O.A., Boragine D.M., Chen Y., Palzkill T. (2021). KPC-2 β-lactamase enables carbapenem antibiotic resistance through fast deacylation of the covalent intermediate. J. Biol. Chem..

[64] Furey I.M., Mehta S.C., Sankaran B., Hu L., Prasad B.V.V., Palzkill T. (2021). Local interactions with the Glu166 base and the conformation of an active site loop play key roles in carbapenem hydrolysis by the KPC-2 β-lactamase. J. Biol. Chem..

[65] Hirvonen V.H.A., Spencer J., van der Kamp M.W. (2021). Antimicrobial Resistance Conferred by OXA-48 β-Lactamases: Towards a Detailed Mechanistic Understanding. Antimicrob. Agents Chemother..

[66] Mitchell J.M., June C.M., Baggett V.L., Lowe B.C., Ruble J.F., Bonomo R.A., Leonard D.A., Powers R.A. (2022). Conformational flexibility in carbapenem hydrolysis drives substrate specificity of the class D carbapenemase OXA-24/40. J. Biol. Chem..

[67] Papp-Wallace K.M., Kumar V., Zeiser E.T., Becka S.A., van den Akker F. (2019). Structural Analysis of The OXA-48 Carbapenemase Bound to A “Poor” Carbapenem Substrate, Doripenem. Antibiotics.

[68] Stewart N.K., Smith C.A., Antunes N.T., Toth M., Vakulenko S.B. (2019). Role of the Hydrophobic Bridge in the Carbapenemase Activity of Class D β-Lactamases. Antimicrob. Agents Chemother..

[69] Lohans C.T., van Groesen E., Kumar K., Tooke C.L., Spencer J., Paton R.S., Brem J., Schofield C.J. (2018). A New Mechanism for β-Lactamases: Class D Enzymes Degrade 1β-Methyl Carbapenems through Lactone Formation. Angew. Chem. Int. Ed. Engl..

[70] Hirvonen V.H.A., Weizmann T.M., Mulholland A.J., Spencer J., van der Kamp M.W. (2022). Multiscale Simulations Identify Origins of Differential Carbapenem Hydrolysis by the OXA-48 β-Lactamase. ACS Catal..

[71] Pemberton O.A., Tsivkovski R., Totrov M., Lomovskaya O., Chen Y. (2020). Structural Basis and Binding Kinetics of Vaborbactam in Class A β-Lactamase Inhibition. Antimicrob. Agents Chemother..

[72] Krishnan N.P., Nguyen N.Q., Papp-Wallace K.M., Bonomo R.A., van den Akker F. (2015). Inhibition of Klebsiella β-Lactamases (SHV-1 and KPC-2) by Avibactam: A Structural Study. PLoS ONE.

[73] Pemberton O.A., Noor R.E., Kumar M.V.V., Sanishvili R., Kemp M.T., Kearns F.L., Woodcock H.L., Gelis I., Chen Y. (2020). Mechanism of proton transfer in class A β-lactamase catalysis and inhibition by avibactam. Proc. Natl. Acad. Sci. USA.

[74] Choi H., Paton R.S., Park H., Schofield C.J. (2016). Investigations on recyclisation and hydrolysis in avibactam mediated serine β-lactamase inhibition. Org. Biomol. Chem..

[75] Papp-Wallace K.M., Barnes M.D., Alsop J., Taracila M.A., Bethel C.R., Becka S.A., van Duin D., Kreiswirth B.N., Kaye K.S., Bonomo R.A. (2018). Relebactam Is a Potent Inhibitor of the KPC-2 β-Lactamase and Restores Imipenem Susceptibility in KPC-Producing Enterobacteriaceae. Antimicrob. Agents Chemother..

[76] Tooke C.L., Hinchliffe P., Bonomo R.A., Schofield C.J., Mulholland A.J., Spencer J. (2021). Natural variants modify Klebsiella pneumoniae carbapenemase (KPC) acyl-enzyme conformational dynamics to extend antibiotic resistance. J. Biol. Chem..

[77] Alsenani T.A., Viviani S.L., Kumar V., Taracila M.A., Bethel C.R., Barnes M.D., Papp-Wallace K.M., Shields R.K., Nguyen M.H., Clancy C.J. (2022). Structural Characterization of the D179N and D179Y Variants of KPC-2 β-Lactamase: Omega-Loop Destabilization as a Mechanism of Resistance to Ceftazidime-Avibactam. Antimicrob. Agents Chemother..

[78] Bebrone C., Moali C., Mahy F., Rival S., Docquier J.D., Rossolini G.M., Fastrez J., Pratt R.F., Frere J.M., Galleni M. (2001). CENTA as a chromogenic substrate for studying β-lactamases. Antimicrob. Agents Chemother..

[79] O’Callaghan C.H., Morris A., Kirby S.M., Shingler A.H. (1972). Novel method for detection of β-lactamases by using a chromogenic cephalosporin substrate. Antimicrob. Agents Chemother..

[80] van Berkel S.S., Brem J., Rydzik A.M., Salimraj R., Cain R., Verma A., Owens R.J., Fishwick C.W., Spencer J., Schofield C.J. (2013). Assay platform for clinically relevant metallo-β-lactamases. J. Med. Chem..

[81] Seidel S.A., Dijkman P.M., Lea W.A., van den Bogaart G., Jerabek-Willemsen M., Lazic A., Joseph J.S., Srinivasan P., Baaske P., Simeonov A. (2013). Microscale thermophoresis quantifies biomolecular interactions under previously challenging conditions. Methods.

[82] Christopeit T., Carlsen T.J., Helland R., Leiros H.K. (2015). Discovery of Novel Inhibitor Scaffolds against the Metallo-β-lactamase VIM-2 by Surface Plasmon Resonance (SPR) Based Fragment Screening. J. Med. Chem..

[83] King A.M., Reid-Yu S.A., Wang W., King D.T., De Pascale G., Strynadka N.C., Walsh T.R., Coombes B.K., Wright G.D. (2014). Aspergillomarasmine A overcomes metallo-β-lactamase antibiotic resistance. Nature.

[84] Sychantha D., Rotondo C.M., Tehrani K., Martin N.I., Wright G.D. (2021). Aspergillomarasmine A inhibits metallo-β-lactamases by selectively sequestering Zn(2). J. Biol. Chem..

[85] Koteva K., Sychantha D., Rotondo C.M., Hobson C., Britten J.F., Wright G.D. (2022). Three-Dimensional Structure and Optimization of the Metallo-β-Lactamase Inhibitor Aspergillomarasmine A. ACS Omega.

[86] Taylor D.M., Anglin J., Park S., Ucisik M.N., Faver J.C., Simmons N., Jin Z., Palaniappan M., Nyshadham P., Li F. (2020). Identifying Oxacillinase-48 Carbapenemase Inhibitors Using DNA-Encoded Chemical Libraries. ACS Infect. Dis..

[87] Jeffs M.A., Gray R.A.V., Sheth P.M., Lohans C.T. (2023). Development of a whole-cell biosensor for β-lactamase inhibitor discovery. Chem. Commun. (Camb).

[88] Yin J., Zhu Y., Liang Y., Luo Y., Lou J., Hu X., Meng Q., Zhu T., Yu Z. (2022). Development of Whole-Cell Biosensors for Screening of Peptidoglycan-Targeting Antibiotics in a Gram-Negative Bacterium. Appl. Environ. Microbiol..

[89] Lyu J., Wang S., Balius T.E., Singh I., Levit A., Moroz Y.S., O’Meara M.J., Che T., Algaa E., Tolmachova K. (2019). Ultra-large library docking for discovering new chemotypes. Nature.

[90] Spyrakis F., Santucci M., Maso L., Cross S., Gianquinto E., Sannio F., Verdirosa F., De Luca F., Docquier J.D., Cendron L. (2020). Virtual screening identifies broad-spectrum β-lactamase inhibitors with activity on clinically relevant serine- and metallo-carbapenemases. Sci. Rep..

[91] Caburet J., Boucherle B., Bourdillon S., Simoncelli G., Verdirosa F., Docquier J.D., Moreau Y., Krimm I., Crouzy S., Peuchmaur M. (2022). A fragment-based drug discovery strategy applied to the identification of NDM-1 β-lactamase inhibitors. Eur. J. Med. Chem..

[92] Li G.B., Abboud M.I., Brem J., Someya H., Lohans C.T., Yang S.Y., Spencer J., Wareham D.W., McDonough M.A., Schofield C.J. (2017). NMR-filtered virtual screening leads to non-metal chelating metallo-β-lactamase inhibitors. Chem. Sci..

[93] Li X., Cai Y., Xia Q., Liao Y., Qin R. (2023). Antibacterial sensitizers from natural plants: A powerful weapon against methicillin-resistant Staphylococcus aureus. Front. Pharmacol..

[94] Shin J., Prabhakaran V.S., Kim K.S. (2018). The multi-faceted potential of plant-derived metabolites as antimicrobial agents against multidrug-resistant pathogens. Microb. Pathog..

[95] He Y., Zhou S., Sun W., Li Q., Wang J., Zhang J. (2022). Emerione A, a novel fungal metabolite as an inhibitor of New Delhi metallo-β-lactamase 1, restores carbapenem susceptibility in carbapenem-resistant isolates. J. Glob. Antimicrob. Resist..

[96] Guo Y., Yang Y., Xu X., Li L., Zhou Y., Jia G., Wei L., Yu Q., Wang J. (2022). Metallo-β-lactamases inhibitor fisetin attenuates meropenem resistance in NDM-1-producing *Escherichia coli*. Eur. J. Med. Chem..

[97] Benin B.M., Hillyer T., Crugnale A.S., Fulk A., Thomas C.A., Crowder M.W., Smith M.A., Shin W.S. (2023). Taxifolin as a Metallo-β-Lactamase Inhibitor in Combination with Augmentin against Verona Imipenemase 2 Expressing Pseudomonas aeruginosa. Microorganisms.

[98] Elfaky M.A., El-Halawany A.M., Koshak A.E., Alshali K.Z., El-Araby M.E., Khayat M.T., Abdallah H.M. (2020). Bioassay Guided Isolation and Docking Studies of a Potential β-Lactamase Inhibitor from Clutia myricoides. Molecules.

[99] CDC & FDA Antimicrobial Resistance (AR) Isolate Bank. https://www.cdc.gov/drugresistance/resistance-bank/index.html.

[100] National Center for Advancing Translational Sciences Compound Management Capabilites. https://ncats.nih.gov/research/research-activities/compound-management.

[101] Gonzalez-Bello C., Rodriguez D., Pernas M., Rodriguez A., Colchon E. (2020). β-Lactamase Inhibitors To Restore the Efficacy of Antibiotics against Superbugs. J. Med. Chem..

[102] Davies D.T., Everett M. (2021). Designing Inhibitors of β-Lactamase Enzymes to Overcome Carbapenem Resistance in Gram-Negative Bacteria. Acc. Chem. Res..

[103] Li X., Zhao J., Zhang B., Duan X., Jiao J., Wu W., Zhou Y., Wang H. (2022). Drug development concerning metallo-β-lactamases in gram-negative bacteria. Front. Microbiol..

[104] Chen C., Oelschlaeger P., Wang D., Xu H., Wang Q., Wang C., Zhao A., Yang K.W. (2022). Structure and Mechanism-Guided Design of Dual Serine/Metallo-Carbapenemase Inhibitors. J. Med. Chem..

[105] Rojas L.J., Taracila M.A., Papp-Wallace K.M., Bethel C.R., Caselli E., Romagnoli C., Winkler M.L., Spellberg B., Prati F., Bonomo R.A. (2016). Boronic Acid Transition State Inhibitors Active against KPC and Other Class A β-Lactamases: Structure-Activity Relationships as a Guide to Inhibitor Design. Antimicrob. Agents Chemother..

[106] Zhou J., Stapleton P., Xavier-Junior F.H., Schatzlein A., Haider S., Healy J., Wells G. (2022). Triazole-substituted phenylboronic acids as tunable lead inhibitors of KPC-2 antibiotic resistance. Eur. J. Med. Chem..

[107] Introvigne M.L., Beardsley T.J., Fernando M.C., Leonard D.A., Wallar B.J., Rudin S.D., Taracila M.A., Rather P.N., Colquhoun J.M., Song S. (2023). Sulfonamidoboronic Acids as “Cross-Class” Inhibitors of an Expanded-Spectrum Class C Cephalosporinase, ADC-33, and a Class D Carbapenemase, OXA-24/40: Strategic Compound Design to Combat Resistance in Acinetobacter baumannii. Antibiotics.

[108] Chen Y., Shoichet B., Bonnet R. (2005). Structure, function, and inhibition along the reaction coordinate of CTX-M β-lactamases. J. Am. Chem. Soc..

[109] Perbandt M., Werner N., Prester A., Rohde H., Aepfelbacher M., Hinrichs W., Betzel C. (2022). Structural basis to repurpose boron-based proteasome inhibitors Bortezomib and Ixazomib as β-lactamase inhibitors. Sci. Rep..

[110] Bouchet F., Atze H., Fonvielle M., Edoo Z., Arthur M., Etheve-Quelquejeu M., Iannazzo L. (2020). Diazabicyclooctane Functionalization for Inhibition of β-Lactamases from Enterobacteria. J. Med. Chem..

[111] Taylor D.M., Anglin J., Hu L., Wang L., Sankaran B., Wang J., Matzuk M.M., Prasad B.V.V., Palzkill T. (2021). Unique Diacidic Fragments Inhibit the OXA-48 Carbapenemase and Enhance the Killing of *Escherichia coli* Producing OXA-48. ACS Infect. Dis..

[112] Pemberton O.A., Zhang X., Nichols D.A., DeFrees K., Jaishankar P., Bonnet R., Adams J., Shaw L.N., Renslo A.R., Chen Y. (2018). Antibacterial Spectrum of a Tetrazole-Based Reversible Inhibitor of Serine β-Lactamases. Antimicrob. Agents Chemother..

[113] Hinchliffe P., Tanner C.A., Krismanich A.P., Labbe G., Goodfellow V.J., Marrone L., Desoky A.Y., Calvopina K., Whittle E.E., Zeng F. (2018). Structural and Kinetic Studies of the Potent Inhibition of Metallo-β-lactamases by 6-Phosphonomethylpyridine-2-carboxylates. Biochemistry.

[114] Chen A.Y., Thomas P.W., Stewart A.C., Bergstrom A., Cheng Z., Miller C., Bethel C.R., Marshall S.H., Credille C.V., Riley C.L. (2017). Dipicolinic Acid Derivatives as Inhibitors of New Delhi Metallo-β-lactamase-1. J. Med. Chem..

[115] Li R., Su H., Chen W., Yan Y.H., Zhou C., Mou L., Yang H., Qian S., Wang Z., Yang L. (2022). Design, Synthesis, and Biological Evaluation of New 1H-Imidazole-2-Carboxylic Acid Derivatives as Metallo-β-Lactamase Inhibitors. Bioorg. Med. Chem..

[116] Wachino J.I., Jin W., Kimura K., Kurosaki H., Sato A., Arakawa Y. (2020). Sulfamoyl Heteroarylcarboxylic Acids as Promising Metallo-β-Lactamase Inhibitors for Controlling Bacterial Carbapenem Resistance. mBio.

[117] Brem J., Panduwawala T., Hansen J.U., Hewitt J., Liepins E., Donets P., Espina L., Farley A.J.M., Shubin K., Campillos G.G. (2022). Imitation of β-lactam binding enables broad-spectrum metallo-β-lactamase inhibitors. Nat. Chem..

[118] Kaya C., Konstantinovic J., Kany A.M., Andreas A., Kramer J.S., Brunst S., Weizel L., Rotter M.J., Frank D., Yahiaoui S. (2022). N-Aryl Mercaptopropionamides as Broad-Spectrum Inhibitors of Metallo-β-Lactamases. J. Med. Chem..

[119] Li J.Q., Gao H., Zhai L., Sun L.Y., Chen C., Chigan J.Z., Ding H.H., Yang K.W. (2021). Dipyridyl-substituted thiosemicarbazone as a potent broad-spectrum inhibitor of metallo-β-lactamases. Bioorg. Med. Chem..

[120] Christopeit T., Albert A., Leiros H.S. (2016). Discovery of a novel covalent non-β-lactam inhibitor of the metallo-β-lactamase NDM-1. Bioorg. Med. Chem..

[121] Thomas P.W., Cho E.J., Bethel C.R., Smisek T., Ahn Y.C., Schroeder J.M., Thomas C.A., Dalby K.N., Beckham J.T., Crowder M.W. (2022). Discovery of an Effective Small-Molecule Allosteric Inhibitor of New Delhi Metallo-β-lactamase (NDM). ACS Infect. Dis..

[122] Hecker S.J., Reddy K.R., Lomovskaya O., Griffith D.C., Rubio-Aparicio D., Nelson K., Tsivkovski R., Sun D., Sabet M., Tarazi Z. (2020). Discovery of Cyclic Boronic Acid QPX7728, an Ultrabroad-Spectrum Inhibitor of Serine and Metallo-β-lactamases. J. Med. Chem..

[123] Lomovskaya O., Tsivkovski R., Sun D., Reddy R., Totrov M., Hecker S., Griffith D., Loutit J., Dudley M. (2021). QPX7728, An Ultra-Broad-Spectrum B-Lactamase Inhibitor for Intravenous and Oral Therapy: Overview of Biochemical and Microbiological Characteristics. Front. Microbiol..

[124] Wang Y.L., Liu S., Yu Z.J., Lei Y., Huang M.Y., Yan Y.H., Ma Q., Zheng Y., Deng H., Sun Y. (2019). Structure-Based Development of (1-(3’-Mercaptopropanamido)methyl)boronic Acid Derived Broad-Spectrum, Dual-Action Inhibitors of Metallo- and Serine-β-lactamases. J. Med. Chem..

[125] Santucci M., Spyrakis F., Cross S., Quotadamo A., Farina D., Tondi D., De Luca F., Docquier J.D., Prieto A.I., Ibacache C. (2017). Computational and biological profile of boronic acids for the detection of bacterial serine- and metallo-β-lactamases. Sci. Rep..

[126] Romero E., Oueslati S., Benchekroun M., D’Hollander A.C.A., Ventre S., Vijayakumar K., Minard C., Exilie C., Tlili L., Retailleau P. (2021). Azetidinimines as a novel series of non-covalent broad-spectrum inhibitors of β-lactamases with submicromolar activities against carbapenemases KPC-2 (class A), NDM-1 (class B) and OXA-48 (class D). Eur. J. Med. Chem..

[127] Pemberton O.A., Jaishankar P., Akhtar A., Adams J.L., Shaw L.N., Renslo A.R., Chen Y. (2019). Heteroaryl Phosphonates as Noncovalent Inhibitors of Both Serine- and Metallocarbapenemases. J. Med. Chem..

[128] Rajavel M., Kumar V., Nguyen H., Wyatt J., Marshall S.H., Papp-Wallace K.M., Deshpande P., Bhavsar S., Yeole R., Bhagwat S. (2021). Structural Characterization of Diazabicyclooctane β-Lactam “Enhancers” in Complex with Penicillin-Binding Proteins PBP2 and PBP3 of Pseudomonas aeruginosa. mBio.

[129] Papp-Wallace K.M., Bonomo R.A. (2016). New β-Lactamase Inhibitors in the Clinic. Infect. Dis. Clin. N. Am..

[130] Cahill S.T., Cain R., Wang D.Y., Lohans C.T., Wareham D.W., Oswin H.P., Mohammed J., Spencer J., Fishwick C.W., McDonough M.A. (2017). Cyclic Boronates Inhibit All Classes of β-Lactamases. Antimicrob. Agents Chemother..

[131] Tondi D., Venturelli A., Bonnet R., Pozzi C., Shoichet B.K., Costi M.P. (2014). Targeting class A and C serine β-lactamases with a broad-spectrum boronic acid derivative. J. Med. Chem..

[132] Ness S., Martin R., Kindler A.M., Paetzel M., Gold M., Jensen S.E., Jones J.B., Strynadka N.C. (2000). Structure-based design guides the improved efficacy of deacylation transition state analogue inhibitors of TEM-1 β-Lactamase(,). Biochemistry.

[133] Stachyra T., Levasseur P., Pechereau M.C., Girard A.M., Claudon M., Miossec C., Black M.T. (2009). In vitro activity of the β-lactamase inhibitor NXL104 against KPC-2 carbapenemase and Enterobacteriaceae expressing KPC carbapenemases. J. Antimicrob. Chemother..

[134] Livermore D.M., Mushtaq S., Warner M., Miossec C., Woodford N. (2008). NXL104 combinations versus Enterobacteriaceae with CTX-M extended-spectrum β-lactamases and carbapenemases. J. Antimicrob. Chemother..

[135] Coleman K. (2011). Diazabicyclooctanes (DBOs): A potent new class of non-β-lactam β-lactamase inhibitors. Curr. Opin. Microbiol..

[136] Shapiro A.B., Moussa S.H., McLeod S.M., Durand-Reville T., Miller A.A. (2021). Durlobactam, a New Diazabicyclooctane β-Lactamase Inhibitor for the Treatment of Acinetobacter Infections in Combination With Sulbactam. Front. Microbiol..

[137] Durand-Reville T.F., Guler S., Comita-Prevoir J., Chen B., Bifulco N., Huynh H., Lahiri S., Shapiro A.B., McLeod S.M., Carter N.M. (2017). ETX2514 is a broad-spectrum β-lactamase inhibitor for the treatment of drug-resistant Gram-negative bacteria including Acinetobacter baumannii. Nat. Microbiol..

[138] Nichols D.A., Hargis J.C., Sanishvili R., Jaishankar P., Defrees K., Smith E.W., Wang K.K., Prati F., Renslo A.R., Woodcock H.L. (2015). Ligand-Induced Proton Transfer and Low-Barrier Hydrogen Bond Revealed by X-ray Crystallography. J. Am. Chem. Soc..

[139] Mojica M.F., Rossi M.A., Vila A.J., Bonomo R.A. (2022). The urgent need for metallo-β-lactamase inhibitors: An unattended global threat. Lancet Infect. Dis..

[140] Lomovskaya O., Tsivkovski R., Totrov M., Dressel D., Castanheira M., Dudley M. (2023). New boronate drugs and evolving NDM-mediated β-lactam resistance. Antimicrob. Agents Chemother..

[141] Yan Y.H., Zhang T.T., Li R., Wang S.Y., Wei L.L., Wang X.Y., Zhu K.R., Li S.R., Liang G.Q., Yang Z.B. (2023). Discovery of 2-Aminothiazole-4-carboxylic Acids as Broad-Spectrum Metallo-β-lactamase Inhibitors by Mimicking Carbapenem Hydrolysate Binding. J. Med. Chem..

[142] Galdadas I., Qu S., Oliveira A.S.F., Olehnovics E., Mack A.R., Mojica M.F., Agarwal P.K., Tooke C.L., Gervasio F.L., Spencer J. (2021). Allosteric communication in class A β-lactamases occurs via cooperative coupling of loop dynamics. Elife.

[143] Hellemann E., Nallathambi A., Durrant J.D. (2023). Allosteric inhibition of TEM-1 β lactamase: Microsecond molecular dynamics simulations provide mechanistic insights. Protein Sci..

[144] Liu B., Trout R.E.L., Chu G.H., McGarry D., Jackson R.W., Hamrick J.C., Daigle D.M., Cusick S.M., Pozzi C., De Luca F. (2020). Discovery of Taniborbactam (VNRX-5133): A Broad-Spectrum Serine- and Metallo-β-lactamase Inhibitor for Carbapenem-Resistant Bacterial Infections. J. Med. Chem..

[145] Cendron L., Quotadamo A., Maso L., Bellio P., Montanari M., Celenza G., Venturelli A., Costi M.P., Tondi D. (2019). X-ray Crystallography Deciphers the Activity of Broad-Spectrum Boronic Acid β-Lactamase Inhibitors. ACS Med. Chem. Lett..

[146] Meroueh S.O., Minasov G., Lee W., Shoichet B.K., Mobashery S. (2003). Structural aspects for evolution of β-lactamases from penicillin-binding proteins. J. Am. Chem. Soc..

[147] Asli A., Brouillette E., Krause K.M., Nichols W.W., Malouin F. (2016). Distinctive Binding of Avibactam to Penicillin-Binding Proteins of Gram-Negative and Gram-Positive Bacteria. Antimicrob. Agents Chemother..

[148] Edoo Z., Iannazzo L., Compain F., Li de la Sierra Gallay I., van Tilbeurgh H., Fonvielle M., Bouchet F., Le Run E., Mainardi J.L., Arthur M. (2018). Synthesis of Avibactam Derivatives and Activity on β-Lactamases and Peptidoglycan Biosynthesis Enzymes of Mycobacteria. Chemistry.

[149] Levy N., Bruneau J.M., Le Rouzic E., Bonnard D., Le Strat F., Caravano A., Chevreuil F., Barbion J., Chasset S., Ledoussal B. (2019). Structural Basis for *E. coli* Penicillin Binding Protein (PBP) 2 Inhibition, a Platform for Drug Design. J. Med. Chem..

[150] Moya B., Barcelo I.M., Bhagwat S., Patel M., Bou G., Papp-Wallace K.M., Bonomo R.A., Oliver A. (2017). WCK 5107 (Zidebactam) and WCK 5153 Are Novel Inhibitors of PBP2 Showing Potent “β-Lactam Enhancer” Activity against Pseudomonas aeruginosa, Including Multidrug-Resistant Metallo-β-Lactamase-Producing High-Risk Clones. Antimicrob. Agents Chemother..

[151] Kumar V., Viviani S.L., Ismail J., Agarwal S., Bonomo R.A., van den Akker F. (2021). Structural analysis of the boronic acid β-lactamase inhibitor vaborbactam binding to Pseudomonas aeruginosa penicillin-binding protein 3. PLoS ONE.

[152] Newman H., Krajnc A., Bellini D., Eyermann C.J., Boyle G.A., Paterson N.G., McAuley K.E., Lesniak R., Gangar M., Von Delft F. (2021). High-Throughput Crystallography Reveals Boron-Containing Inhibitors of a Penicillin-Binding Protein with Di- And Tricovalent Binding Modes. J. Med. Chem..

[153] Zervosen A., Bouillez A., Herman A., Amoroso A., Joris B., Sauvage E., Charlier P., Luxen A. (2012). Synthesis and evaluation of boronic acids as inhibitors of Penicillin Binding Proteins of classes A, B and C. Bioorg. Med. Chem..

[154] Łȩski T.A., Tomasz A. (2005). Role of penicillin-binding protein 2 (PBP2) in the antibiotic susceptibility and cell wall cross-linking of Staphylococcus aureus: Evidence for the cooperative functioning of PBP2, PBP4, and PBP2A. J. Bacteriol..

[155] Fishovitz J., Hermoso J.A., Chang M., Mobashery S. (2014). Penicillin-binding protein 2a of methicillin-resistant Staphylococcus aureus. IUBMB Life.

[156] Srisuknimit V., Qiao Y., Schaefer K., Kahne D., Walker S. (2017). Peptidoglycan Cross-Linking Preferences of Staphylococcus aureus Penicillin-Binding Proteins Have Implications for Treating MRSA Infections. J. Am. Chem. Soc..

[157] Mahasenan K.V., Molina R., Bouley R., Batuecas M.T., Fisher J.F., Hermoso J.A., Chang M., Mobashery S. (2017). Conformational Dynamics in Penicillin-Binding Protein 2a of Methicillin-Resistant Staphylococcus aureus, Allosteric Communication Network and Enablement of Catalysis. J. Am. Chem. Soc..

[158] Otero L.H., Rojas-Altuve A., Llarrull L.I., Carrasco-Lopez C., Kumarasiri M., Lastochkin E., Fishovitz J., Dawley M., Hesek D., Lee M. (2013). How allosteric control of Staphylococcus aureus penicillin binding protein 2a enables methicillin resistance and physiological function. Proc. Natl. Acad. Sci. USA.

[159] Lim D., Strynadka N.C. (2002). Structural basis for the β lactam resistance of PBP2a from methicillin-resistant Staphylococcus aureus. Nat. Struct. Biol..

[160] Moon T.M., D’Andrea E.D., Lee C.W., Soares A., Jakoncic J., Desbonnet C., Garcia-Solache M., Rice L.B., Page R., Peti W. (2018). The structures of penicillin-binding protein 4 (PBP4) and PBP5 from Enterococci provide structural insights into β-lactam resistance. J. Biol. Chem..

[161] Hunashal Y., Kumar G.S., Choy M.S., D’Andrea E.D., Da Silva Santiago A., Schoenle M.V., Desbonnet C., Arthur M., Rice L.B., Page R. (2023). Molecular basis of β-lactam antibiotic resistance of ESKAPE bacterium E. faecium Penicillin Binding Protein PBP5. Nat. Commun..

[162] Grebe T., Hakenbeck R. (1996). Penicillin-binding proteins 2b and 2x of Streptococcus pneumoniae are primary resistance determinants for difierent classes of β-lactam antibiotics. Antimicrob. Agents Chemother..

[163] Tsui H.C.T., Boersma M.J., Vella S.A., Kocaoglu O., Kuru E., Peceny J.K., Carlson E.E., Vannieuwenhze M.S., Brun Y.V., Shaw S.L. (2014). Pbp2x localizes separately from Pbp2b and other peptidoglycan synthesis proteins during later stages of cell division of Streptococcus pneumoniae D39. Mol. Microbiol..

[164] Gordon E., Mouz N., Duée E., Dideberg O. (2000). The crystal structure of the penicillin-binding protein 2x from Streptococcus pneumoniae and its acyl-enzyme form: Implication in drug resistance. J. Mol. Biol..

[165] Sacco M.D., Wang S., Adapa S.R., Zhang X., Lewandowski E.M., Gongora M.V., Keramisanou D., Atlas Z.D., Townsend J.A., Gatdula J.R. (2022). A unique class of Zn^2+^-binding serine-based PBPs underlies cephalosporin resistance and sporogenesis in Clostridioides difficile. Nat. Commun..

[166] Smith J.D., Kumarasiri M., Zhang W., Hesek D., Lee M., Toth M., Vakulenko S., Fisher J.F., Mobashery S., Chen Y. (2013). Structural analysis of the role of Pseudomonas aeruginosa penicillin-binding protein 5 in β-lactam resistance. Antimicrob. Agents Chemother..

[167] Kumar G., Galanis C., Batchelder H.R., Townsend C.A., Lamichhane G. (2022). Penicillin Binding Proteins and β-Lactamases of Mycobacterium tuberculosis: Reexamination of the Historical Paradigm. mSphere.

[168] Both D., Steiner E.M., Stadler D., Lindqvist Y., Schnell R., Schneider G. (2013). Structure of LdtMt2, an L,D-transpeptidase from Mycobacterium tuberculosis. Acta Crystallogr. D Biol. Crystallogr..

[169] Gupta R., Lavollay M., Mainardi J.L., Arthur M., Bishai W.R., Lamichhane G. (2010). The Mycobacterium tuberculosis gene, ldtMt2, encodes a non-classical transpeptidase required for virulence and resistance to amoxicillin. Nat. Med..

[170] Micelli C., Dai Y., Raustad N., Isberg R.R., Dowson C.G., Lloyd A.J., Geisinger E., Crow A., Roper D.I. (2023). A conserved zinc-binding site in Acinetobacter baumannii PBP2 required for elongasome-directed bacterial cell shape. Proc. Natl. Acad. Sci. USA.

[171] Chen W., Zhang Y.M., Davies C. (2017). Penicillin-Binding Protein 3 Is Essential for Growth of Pseudomonas aeruginosa. Antimicrob. Agents Chemother..

[172] Sacco M.D., Kroeck K.G., Trent Kemp M., Zhang X., Andrews L.D., Chen Y. (2020). Influence of the α-Methoxy Group on the Reaction of Temocillin with Pseudomonas aeruginosa PBP3 and CTX-M-14 β-Lactamase. Antimicrob. Agents Chemother..

[173] Kumar V., Tang C., Bethel C.R., Papp-Wallace K.M., Wyatt J., Desarbre E., Bonomo R.A., van den Akker F. (2020). Structural Insights into Ceftobiprole Inhibition of Pseudomonas aeruginosa Penicillin-Binding Protein 3. Antimicrob. Agents Chemother..

[174] Sharifzadeh S., Dempwolff F., Kearns D.B., Carlson E.E. (2020). Harnessing β-Lactam Antibiotics for Illumination of the Activity of Penicillin-Binding Proteins in Bacillus subtilis. ACS Chem. Biol..

[175] Godinez W.J., Chan H., Hossain I., Li C., Ranjitkar S., Rasper D., Simmons R.L., Zhang X., Feng B.Y. (2019). Morphological Deconvolution of Β-Lactam Polyspecificity in *E. coli*. ACS Chem. Biol..

[176] Sayed A.R.M., Shah N.R., Basso K.B., Kamat M., Jiao Y., Moya B., Sutaria D.S., Lang Y., Tao X., Liu W. (2020). First Penicillin-Binding Protein Occupancy Patterns for 15 β-Lactams and β-Lactamase Inhibitors in Mycobacterium abscessus. Antimicrob. Agents Chemother..

[177] Lopez-Arguello S., Montaner M., Marmol-Salvador A., Velazquez-Escudero A., Docobo-Perez F., Oliver A., Moya B. (2023). Penicillin-Binding Protein Occupancy Dataset for 18 β-Lactams and 4 β-Lactamase Inhibitors in Neisseria gonorrhoeae. Microbiol. Spectr..

[178] Kim T.H., Tao X., Moya B., Jiao Y., Basso K.B., Zhou J., Lang Y., Sutaria D.S., Zavascki A.P., Barth A.L. (2020). Novel Cassette Assay To Quantify the Outer Membrane Permeability of Five β-Lactams Simultaneously in Carbapenem-Resistant Klebsiella pneumoniae and Enterobacter cloacae. mBio.

[179] Lang Y., Shah N.R., Tao X., Reeve S.M., Zhou J., Moya B., Sayed A.R.M., Dharuman S., Oyer J.L., Copik A.J. (2021). Combating Multidrug-Resistant Bacteria by Integrating a Novel Target Site Penetration and Receptor Binding Assay Platform Into Translational Modeling. Clin. Pharmacol. Ther..

[180] Shirley J.D., Nauta K.M., Carlson E.E. (2022). Live-Cell Profiling of Penicillin-Binding Protein Inhibitors in *Escherichia coli* MG1655. ACS Infect. Dis..

[181] Montaner M., Lopez-Arguello S., Oliver A., Moya B. (2023). PBP Target Profiling by β-Lactam and β-Lactamase Inhibitors in Intact Pseudomonas aeruginosa: Effects of the Intrinsic and Acquired Resistance Determinants on the Periplasmic Drug Availability. Microbiol. Spectr..

[182] Gonzales P.R., Pesesky M.W., Bouley R., Ballard A., Biddy B.A., Suckow M.A., Wolter W.R., Schroeder V.A., Burnham C.A., Mobashery S. (2015). Synergistic, collaterally sensitive β-lactam combinations suppress resistance in MRSA. Nat. Chem. Biol..

[183] Jiao Y., Moya B., Chen M.J., Zavascki A.P., Tsai H., Tao X., Sutaria D.S., Louie A., Boyce J.D., Deveson Lucas D. (2019). Comparable Efficacy and Better Safety of Double β-Lactam Combination Therapy versus β-Lactam plus Aminoglycoside in Gram-Negative Bacteria in Randomized, Controlled Trials. Antimicrob. Agents Chemother..

[184] Smith N.M., Lenhard J.R., Boissonneault K.R., Landersdorfer C.B., Bulitta J.B., Holden P.N., Forrest A., Nation R.L., Li J., Tsuji B.T. (2020). Using machine learning to optimize antibiotic combinations: Dosing strategies for meropenem and polymyxin B against carbapenem-resistant Acinetobacter baumannii. Clin. Microbiol. Infect. Off. Publ. Eur. Soc. Clin. Microbiol. Infect. Dis..

[185] Hogan A.M., Rahman A., Motnenko A., Natarajan A., Maydaniuk D.T., Leon B., Batun Z., Palacios A., Bosch A., Cardona S.T. (2023). Profiling cell envelope-antibiotic interactions reveals vulnerabilities to β-lactams in a multidrug-resistant bacterium. Nat. Commun..

[186] Zhao G., Meier T.I., Kahl S.D., Gee K.R., Blaszczak L.C. (1999). BOCILLIN FL, a Sensitive and Commercially Available Reagent for Detection of Penicillin-Binding Proteins. Antimicrob. Agents Chemother..

[187] Shapiro A.B., Gu R.F., Gao N., Livchak S., Thresher J. (2013). Continuous fluorescence anisotropy-based assay of BOCILLIN FL penicillin reaction with penicillin binding protein 3. Anal. Biochem..

[188] Mao W., Wang Y., Qian X., Xia L., Xie H. (2019). A Carbapenem-Based Off-On Fluorescent Probe for Specific Detection of Metallo-β-Lactamase Activities. Chembiochem.

[189] Mao W., Xia L., Xie H. (2017). Detection of Carbapenemase-Producing Organisms with a Carbapenem-Based Fluorogenic Probe. Angew. Chem. Int. Ed. Engl..

[190] June C.M., Vaughan R.M., Ulberg L.S., Bonomo R.A., Witucki L.A., Leonard D.A. (2014). A fluorescent carbapenem for structure function studies of penicillin-binding proteins, β-lactamases, and β-lactam sensors. Anal. Biochem..

[191] Ma C.W., Ng K.K., Yam B.H., Ho P.L., Kao R.Y., Yang D. (2021). Rapid Broad Spectrum Detection of Carbapenemases with a Dual Fluorogenic-Colorimetric Probe. J. Am. Chem. Soc..

[192] Zhao G., Meier T.I., Hoskins J., McAllister K.A. (2000). Identification and Characterization of the Penicillin-Binding Protein 2a of Streptococcus pneumoniae and Its Possible Role in Resistance to β-Lactam Antibiotics. Antimicrob. Agents Chemother..

[193] López-Pérez A., Freischem S., Grimm I., Weiergräber O., Dingley A.J., López-Alberca M.P., Waldmann H., Vollmer W., Kumar K., Vuong C. (2021). Discovery of pyrrolidine-2,3-diones as novel inhibitors of p. Aeruginosa pbp3. Antibiotics.

[194] Adam M., Damblon C., Plaitin B., Christiaens L., Frere J.M. (1990). Chromogenic depsipeptide substrates for β-lactamases and penicillin-sensitive DD-peptidases. Biochem. J..

[195] Pratt R.F., Govardhan C.P. (1984). β-Lactamase-catalyzed hydrolysis of acyclic depsipeptides and acyl transfer to specific amino acid acceptors. Proc. Natl. Acad. Sci. USA.

[196] Gupta R., Al-Kharji N., Alqurafi M.A., Nguyen T.Q., Chai W., Quan P., Malhotra R., Simcox B.S., Mortimer P., Brammer Basta L.A. (2021). Atypically Modified Carbapenem Antibiotics Display Improved Antimycobacterial Activity in the Absence of β-Lactamase Inhibitors. ACS Infect. Dis..

[197] Zandi T.A., Marshburn R.L., Stateler P.K., Brammer Basta L.A. (2019). Phylogenetic and biochemical analyses of mycobacterial L,D-transpeptidases reveal a distinct enzyme class that is preferentially acylated by meropenem. ACS Infect. Dis..

[198] Zandi T.A., Townsend C.A. (2021). Competing off-loading mechanisms of meropenem from an L,D-transpeptidase reduce antibiotic effectiveness. Proc. Natl. Acad. Sci. USA.

[199] Qiao Y., Srisuknimit V., Rubino F., Schaefer K., Ruiz N., Walker S., Kahne D. (2017). Lipid II overproduction allows direct assay of transpeptidase inhibition by β-lactams. Nat. Chem. Biol..

[200] Lv N., Kong Q., Zhang H., Li J. (2021). Discovery of novel Staphylococcus aureus penicillin binding protein 2a inhibitors by multistep virtual screening and biological evaluation. Bioorg. Med. Chem. Lett..

[201] Kulanthaivel L., Jeyaraman J., Biswas A., Subbaraj G.K., Santhoshkumar S. (2018). Identification of potential inhibitors for Penicillinbinding protein (PBP) from *Staphylococcus aureus*. Bioinformation.

[202] Ibrahim M.A.A., Abdeljawaad K.A.A., Abdelrahman A.H.M., Alzahrani O.R., Alshabrmi F.M., Khalaf E., Moustafa M.F., Alrumaihi F., Allemailem K.S., Soliman M.E.S. (2021). Non- β-Lactam Allosteric Inhibitors Target Methicillin-Resistant Staphylococcus aureus: An In Silico Drug Discovery Study. Antibiotics.

[203] Verma A.K., Ahmed S.F., Hossain M.S., Bhojiya A.A., Mathur A., Upadhyay S.K., Srivastava A.K., Vishvakarma N.K., Barik M., Rahaman M.M. (2022). Molecular docking and simulation studies of flavonoid compounds against PBP-2a of methicillin-resistant Staphylococcus aureus. J. Biomol. Struct. Dyn..

[204] Onoabedje E.A., Ibezim A., Okafor S.N., Onoabedje U.S., Okoro U.C. (2016). Oxazin-5-Ones as a Novel Class of Penicillin Binding Protein Inhibitors: Design, Synthesis and Structure Activity Relationship. PLoS ONE.

[205] Sabe V.T., Tolufashe G.F., Ibeji C.U., Maseko S.B., Govender T., Maguire G.E.M., Lamichhane G., Honarparvar B., Kruger H.G. (2019). Identification of potent L,D-transpeptidase 5 inhibitors for Mycobacterium tuberculosis as potential anti-TB leads: Virtual screening and molecular dynamics simulations. J. Mol. Model..

[206] Masumi M., Noormohammadi F., Kianisaba F., Nouri F., Taheri M., Taherkhani A. (2022). Methicillin-Resistant Staphylococcus aureus: Docking-Based Virtual Screening and Molecular Dynamics Simulations to Identify Potential Penicillin-Binding Protein 2a Inhibitors from Natural Flavonoids. Int. J. Microbiol..

[207] Nandhini P., Gupta P.K., Mahapatra A.K., Das A.P., Agarwal S.M., Mickymaray S., Alothaim A.S., Rajan M. (2023). In-Silico molecular screening of natural compounds as a potential therapeutic inhibitor for Methicillin-resistant Staphylococcus aureus inhibition. Chem. Biol. Interact..

[208] Qiao Y., Zhang X., He Y., Sun W., Feng W., Liu J., Hu Z., Xu Q., Zhu H., Zhang J. (2018). Aspermerodione, a novel fungal metabolite with an unusual 2,6-dioxabicyclo[2.2.1]heptane skeleton, as an inhibitor of penicillin-binding protein 2a. Sci. Rep..

[209] Turk S., Verlaine O., Gerards T., Živec M., Humljan J., Sosič I., Amoroso A., Zervosen A., Luxen A., Joris B. (2011). New noncovalent inhibitors of penicillin-binding proteins from penicillin-resistant bacteria. PLoS ONE.

[210] Young M., Walsh D.J., Masters E., Gondil V.S., Laskey E., Klaczko M., Awad H., McGrath J., Schwarz E.M., Melander C. (2022). Identification of Staphylococcus aureus Penicillin Binding Protein 4 (PBP4) Inhibitors. Antibiotics.

[211] Stewart N.K., Toth M., Alqurafi M.A., Chai W., Nguyen T.Q., Quan P., Lee M., Buynak J.D., Smith C.A., Vakulenko S.B. (2022). C6 Hydroxymethyl-Substituted Carbapenem MA-1-206 Inhibits the Major Acinetobacter baumannii Carbapenemase OXA-23 by Impeding Deacylation. mBio.

[212] Batchelder H.R., Zandi T.A., Kaushik A., Naik A., Story-Roller E., Maggioncalda E.C., Lamichhane G., Nuermberger E.L., Townsend C.A. (2022). Structure−Activity Relationship of Penem Antibiotic Side Chains Used against Mycobacteria Reveals Highly Active Compounds. ACS Infect. Dis..

[213] Reck F., Bermingham A., Blais J., Capka V., Cariaga T., Casarez A., Colvin R., Dean C.R., Fekete A., Gong W. (2018). Optimization of novel monobactams with activity against carbapenem-resistant Enterobacteriaceae—Identification of LYS228. Bioorg. Med. Chem. Lett..

[214] Fei Z., Wu Q., Li L., Jiang Q., Li B., Chen L., Wang H., Wu B., Wang X., Gao F. (2020). New Synthesis for the Monobactam Antibiotic-LYS228. J. Org. Chem..

[215] Sun Y., Liao X., Huang Z., Xie Y., Liu Y., Ma C., Jiang B., Zhang L., Yan Y., Li G. (2020). Therapeutic Effect and Mechanisms of the Novel Monosulfactam 0073. Antimicrob. Agents Chemother..

[216] Decuyper L., Jukic M., Sosic I., Amoroso A.M., Verlaine O., Joris B., Gobec S., D’Hooghe M. (2020). Synthesis and Penicillin-binding Protein Inhibitory Assessment of Dipeptidic 4-Phenyl-β-lactams from alpha-Amino Acid-derived Imines. Chem. Asian J..

[217] Cherian P.T., Cheramie M.N., Marreddy R.K.R., Fernando D.M., Hurdle J.G., Lee R.E. (2018). New β-lactam—Tetramic acid hybrids show promising antibacterial activities. Bioorg. Med. Chem. Lett..

[218] Halasohoris S.A., Scarff J.M., Pysz L.M., Lembirik S., Lemmon M.M., Biek D., Hannah B., Zumbrun S.D., Panchal R.G. (2021). In vitro and in vivo activity of GT-1, a novel siderophore cephalosporin, and GT-055, a broad-spectrum β-lactamase inhibitor, against biothreat and ESKAPE pathogens. J. Antibiot..

[219] Goldberg J.A., Kumar V., Spencer E.J., Hoyer D., Marshall S.H., Hujer A.M., Hujer K.M., Bethel C.R., Papp-Wallace K.M., Perez F. (2021). A γ-lactam siderophore antibiotic effective against multidrug-resistant Pseudomonas aeruginosa, Klebsiella pneumoniae, and Acinetobacter spp. Eur. J. Med. Chem..

[220] Contreras-Martel C., Amoroso A., Woon E.C., Zervosen A., Inglis S., Martins A., Verlaine O., Rydzik A.M., Job V., Luxen A. (2011). Structure-guided design of cell wall biosynthesis inhibitors that overcome β-lactam resistance in Staphylococcus aureus (MRSA). ACS Chem. Biol..

[221] Durand-Reville T.F., Miller A.A., O’Donnell J.P., Wu X., Sylvester M.A., Guler S., Iyer R., Shapiro A.B., Carter N.M., Velez-Vega C. (2021). Rational design of a new antibiotic class for drug-resistant infections. Nature.

[222] Flanders P.L., Contreras-Martel C., Brown N.W., Shirley J.D., Martins A., Nauta K.N., Dessen A., Carlson E.E., Ambrose E.A. (2022). Combined Structural Analysis and Molecular Dynamics Reveal Penicillin-Binding Protein Inhibition Mode with β-Lactones. ACS Chem. Biol..

[223] O’Daniel P.I., Peng Z., Pi H., Testero S.A., Ding D., Spink E., Leemans E., Boudreau M.A., Yamaguchi T., Schroeder V.A. (2014). Discovery of a New Class of Non-β-lactam Inhibitors of Penicillin-Binding Proteins with Gram-Positive Antibacterial Activity. J. Am. Chem. Soc..

[224] Fisher J.F., Mobashery S. (2023). β-Lactams from the Ocean. Mar. Drugs.

[225] Sharifzadeh S., Boersma M.J., Kocaoglu O., Shokri A., Brown C.L., Shirley J.D., Winkler M.E., Carlson E.E. (2017). Novel Electrophilic Scaffold for Imaging of Essential Penicillin-Binding Proteins in Streptococcus pneumoniae. ACS Chem. Biol..

[226] Brown N.W., Shirley J.D., Marshall A.P., Carlson E.E. (2021). Comparison of Bioorthogonal β-Lactone Activity-Based Probes for Selective Labeling of Penicillin-Binding Proteins. Chembiochem.

[227] Qian Y., Birhanu B.T., Yang J., Ding D., Janardhanan J., Mobashery S., Chang M. (2023). A Potent and Narrow-Spectrum Antibacterial against Clostridioides difficile Infection. J. Med. Chem..

